# Plasmids serve as vehicles and reservoirs of type VI secretion systems

**DOI:** 10.1093/ismeco/ycag069

**Published:** 2026-03-23

**Authors:** María del Mar Quiñonero-Coronel, M Pilar Garcillán-Barcia

**Affiliations:** Instituto de Biomedicina y Biotecnología de Cantabria (IBBTEC, Consejo Superior de Investigaciones Científicas-Universidad de Cantabria), C/ Albert Einstein, 22. PCTCAN. Santander, Cantabria 39011, Spain; Instituto de Biomedicina y Biotecnología de Cantabria (IBBTEC, Consejo Superior de Investigaciones Científicas-Universidad de Cantabria), C/ Albert Einstein, 22. PCTCAN. Santander, Cantabria 39011, Spain

**Keywords:** type VI secretion system, plasmids, horizontal gene transfer

## Abstract

The Type VI secretion system (T6SS) is a major determinant of bacterial competition, yet its dissemination across lineages remains unclear. By analyzing 43 213 plasmids and 29 161 chromosomes, we reveal plasmids as an underestimated reservoir and vehicle for T6SS diversification. We identified 405 complete plasmid-encoded T6SSs and 929 orphan islands containing *hcp, vgrG*, and/or *PAAR* genes, often independent of full systems. Plasmid-encoded T6SSs are biased toward large replicons, frequently megaplasmids, with distinct stability and mobility traits: orphan island plasmids are enriched in conjugation modules, whereas complete systems are associated with partition and toxin-antitoxin maintenance systems. Phylogenomic analyses show that some plasmid lineages stably integrate T6SSs as core traits, while others undergo recurrent acquisition and diversification. Comparative and ancestral analyses indicate pervasive bidirectional transfers between plasmids and chromosomes, with insertion sequences frequently detected in their vicinity. The presence of near-identical homologs across compartments underscores the capacity of plasmids to transcend phylogenetic barriers and propagate these nanoweapons. Together, our results identify plasmids as dual evolutionary actors in T6SS ecology, functioning as short-term vectors for rapid horizontal spread and as long-term reservoirs that foster stabilization and adaptive diversification.

## Graphical Abstract

**Graphical Abstract:**
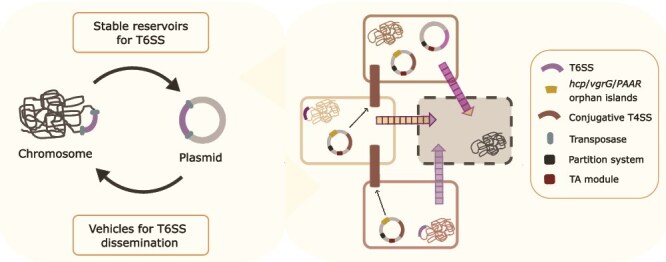

## Introduction

Bacteria have evolved a remarkable array of secretion systems to interact with their environment, ranging from establishing symbiosis to mediating interbacterial competition [1]. Among these, T6SS has emerged as a widespread and versatile nanomachine, capable of delivering toxic effectors into neighboring cells and modulating microbial community structure [2–4]. T6SSs contribute to both antagonistic and cooperative interactions, influencing bacterial survival, adaptation, and ecological fitness [5, 6].

**Figure 1:**
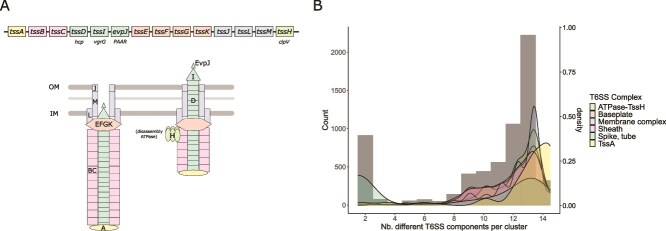
Composition of the detected plasmid-encoded T6SS^i^ clusters. (A) The top panel illustrates the typical components of T6SS genes grouped by functional subcomplexes, without implying a conserved genetic organization, since gene order and synteny can vary substantially among systems. The bottom panel depicts the architecture of T6SS^i^ in its extended (left) and contracted (right) conformations. OM, outer membrane; IM, inner membrane. (B) The abundance of T6SS^i^ genes is shown according to the number of different components detected within the same cluster. Genes included in each T6SS subcomplex are as follows: *tssH* (ATPase); spike and tube (*evpJ/paar, tssI/vgrG,* and *tssD/hcp*); membrane complex (*tssJ, tssL, tssM*); baseplate (*tssE, tssF, tssG, tssK*), sheath (*tssB, tssC*); *tssA*.

At the structural level, the T6SS is a contractile nanomachine evolutionarily related to bacteriophage tail assemblies and sharing architectural similarities with contractile injection systems [7, 8]. It consists of a membrane-anchoring complex (TssJLM) [9], a cytoplasmic baseplate (TssEFGK) [10], and a contractile sheath (TssBC) [11] surrounding an inner tube formed by hexameric Hcp proteins [8] (Fig. 1A). The spike complex, composed of VgrG and sharpened by PAAR-domain proteins [12], punctures target cells and enables the delivery of toxic effectors. Upon sheath contraction, the Hcp tube and associated effectors are propelled outward in a contact-dependent manner (reviewed in [13]). Following firing, the ATPase TssH (also known as ClpV) disassembles the contracted sheath, allowing component recycling and repeated secretion cycles.

Traditionally, T6SSs are encoded in the chromosomes of Gram-negative bacteria [14], where their evolutionary history and functional roles have been extensively studied. However, T6SSs can also be found on plasmids [15], although their functionality has been scarcely tested [16–19]. Because plasmids are inherently mobile and often cross species and genus boundaries [20], plasmid-encoded T6SSs represent potential vectors for the ecological redistribution of bacterial antagonism and cooperation. Their occurrence raises key questions about how mobile genetic elements (MGEs) shape interbacterial competition, niche colonization, and the coevolution of antagonistic systems with plasmid mobility.

Plasmids are central drivers of bacterial evolution, serving as reservoirs of adaptive traits such as antibiotic resistance, metabolic pathways, and virulence factors [21]. Through horizontal gene transfer they enable rapid acquisition of competitive functions [22]. Beyond their role as genetic vehicles, plasmids shape the tempo and mode of bacterial evolution by linking gene mobility with ecological opportunity. The acquisition of T6SSs by plasmids could broaden their ecological roles, promoting the spread of competitive capabilities across bacterial lineages [23]. However, the evolutionary origins, host range, and functional associations of plasmid-borne T6SSs remain poorly understood, as does whether their mobility results in transient acquisitions or enduring ecological strategies. From an ecological and evolutionary perspective, understanding how plasmids mediate the mobilization and stabilization of T6SSs is essential to explain how microbial populations balance horizontal gene flow with competitive stability.

To address these questions, we performed a comprehensive analysis of T6SSs across plasmids and chromosomes from diverse bacterial families. Using integrated network and phylogenomic approaches, we examined their distribution, evolutionary relationships, and associated functional traits. Our results reveal that distinct plasmid lineages specialize in the maintenance and diversification of T6SSs, positioning plasmids not merely as vehicles shuttling systems between genomes but as persistent reservoirs that co-evolve with specific bacterial hosts while occasionally bridging taxonomic boundaries. This lineage-level resolution highlights plasmids as short-term vectors that disseminate T6SS modules across taxa, but also as long-term reservoirs that maintain and diversify these systems within specific lineages, ultimately influencing community composition and ecosystem function.

## Materials and methods

### Genome dataset

Complete genomes were retrieved from the NCBI RefSeq212 database (June 2022), including 29 161 chromosomes and 43 872 plasmids (Supplementary Tables S1–S2). To minimize misclassified entries, plasmids were curated following [24], yielding 43 213 plasmid sequences after filtering.

### T6SS detection and classification

T6SS clusters were identified in chromosomal and plasmid datasets using MacSyFinder v1.0.5 [25] with HMM profiles for subtypes i–iii (T6SS^i^, T6SS^ii^, and T6SS^iii^). Detection parameters required ≥2 distinct T6SS genes within ≤20 intervening genes (–min-genes-required 2; –min-mandatory-genes-required 2; –inter-gene-max-space 20). Clusters with ≥8 components/genes were classified as complete T6SSs and assigned to subtypes via diagnostic markers; others were labeled incomplete or as putative orphan islands if containing *tssD–tssI, tssD–evpJ, tssI–evpJ*, or *tssD–tssI–evpJ* gene pairs. Isolated TssI (VgrG) and TssD (Hcp) proteins were detected using HMMERv3 (hmmsearch; –incE 1e-3, –incdomE 1e-3, ≥50% coverage of profile length). T6SS gene synteny was obtained with Clinker v0.0.31 [26].

### Plasmid classification

Plasmids were assigned to Plasmid Taxonomic Units (PTUs) with COPLA [27]. As previously defined by [28], the threshold for megaplasmid classification was set at ≥5% of the median genome size of the host taxonomic family (Supplementary Table S3), obtained from EZBioCloud [29].

### Plasmid functional annotation

Relaxases and conjugation systems were detected using MOBscan [30] and CONJscan [31]. Antimicrobial resistance (AMR) genes were identified with Staramr [32], using default parameters. Virulence factors were screened with ABRicate against VFDB [33] (https://github.com/tseemann/abricate; BLASTp, e-value <1e-5, ≥50% identity, ≥60% coverage). Insertion sequences were annotated via ISFinder [34]. Recombinases (tyrosine, serine, and resolvase domains; PF00589, PF07058, PF00239) were detected using HMMERv3 (hmmscan; –E 1e-5, –domE 1e-5, –incE 1e-5, –incdomE 1e-5, ≥50% coverage of profile length). Partition systems were searched with hmmsearch (–E 1e-5, –domE 1e-5, –incE 1e-5, –incdomE 1e-5; ≥50% profile coverage) using HMM profiles for type I (PF01656, PF13614, PF18607, NF010259, PF08775, PF02195, PF07506, PF18090, PF08535, PF06613, PF09274), type II (PF06406, PF21523), and type III (PF21493) class components. HMM profiles for the toxins of the toxin-antitoxin (TA) systems contained in TADB3.0 [35] were retrieved using HMMERv3 (hmmscan; –E 1e-5, –domE 1e-5, –incE 1e-5, –incdomE 1e-5, ≥50% sequence coverage). These profiles (Supplementary Table S4) were then used to screen for putative TA modules in the plasmid dataset (hmmsearch; –E 1e-5, –domE 1e-5, –incE 1e-5, –incdomE 1e-5, ≥80% sequence coverage).

### Comparison of GC content

The GC content of plasmids and chromosomes was calculated with in-house scripts in Python v3.13, available at https://github.com/mdmqc/Plasmid_T6SS.

### Phylogenetic analyses

TssC proteins from clusters with ≥10 components were grouped with MMseqs2 15.6f452 [36] at 99% identity and 100% coverage, yielding 2101 representatives. The subtype iv (T6SS^iv^) sheath (WP_012473177.1) from *Amoebophilus asiaticus* 5a2 was included. Alignments were generated with MAFFT v7.271 (–retree 2 –maxiterate 1000) [37], trimmed with TrimAl v1.2 (−automated1) [38] and used to construct a maximum-likelihood (ML) phylogeny in IQ-TREE [39] under the LG + F + R10 model [40], with 1000 ultrafast bootstraps [41]. The tree was rooted with the T4 gp18 sheath subunit. Plasmid diversity within PTUs was assessed using kSNP v3.0 [42] with *Kchooser*-defined k-mers. Trees were reconstructed by maximum parsimony (−core option). All trees were visualized with iTOL v5 [43].

The inference of ancestral states for TssC genomic contexts (chromosomal or plasmid) was carried out with PastML v1.9.33 [44] using ML with the MPPA algorithm under the F81 model.

### Cumulative distribution function

Cumulative distribution functions (CDFs) were generated and visualized with seaborn.ecdfplot (v0.11.0, Python v3.13). They were calculated for plasmid sizes and patristic distances. Patristic distances were computed from the TssC tree using the cophenetic.phylo function in R (package *ape* v5.6–2). For each plasmid-encoded TssC, the closest homolog (plasmid or chromosome) was identified. For clustered but contextually distinct sequences, a distance of zero was assigned.

### Network construction

Monopartite networks of T6SS-encoding plasmid similarity were obtained for pairwise average nucleotide identity values with a 50% plasmid length threshold (ANI_L50_), calculated as described by [20]. Bipartite networks of chromosome–plasmid T6SS transfers were built from homologous protein clusters (HPCs) generated at 99% identity and 100% coverage using MMseqs2 15.6f452 [36]. PTU-specific networks were constructed with AcCNET [45], at 80% identity and 80% coverage. All networks were visualized in Gephi v0.9 with ForceAtlas2 layout.

### Pan-genome and enrichment analyses

PTU pangenomes were constructed with Roary v5.26.2 [46] with default parameters using GFF3 inputs. Gene-T6SS associations were tested with Scoary v1.6.16 [47], with FDR correction (*P* < .05). HPCs were annotated using eggNOG-mapper [48, 49] (e-value ≤1e-3, subject coverage ≥50%). Enrichment of COG categories in T6SS-positive plasmids vs. negative plasmids was tested by Fisher’s exact test, using Benjamini-Hochberg correction and considering categories with adjusted *P* < .05 and odds ratio > 1 as enriched.

### Statistics

Statistical tests were performed in Python v3.13 using the scipy.stats module of the SciPy library. Fisher’s exact tests and one-sided Mann–Whitney U tests were conducted using the functions fisher_exact and scipy.stats.mannwhitneyu.

## Results

### Bacterial genomic landscape of T6SS distribution

To map the genomic distribution of T6SSs, we screened all bacterial chromosomes and plasmids from RefSeq212 using MacSyFinder, retaining genomes encoding ≥ 2 distinct T6SS genes. Among 43 213 plasmids retrieved from the RefSeq dataset prior to any T6SS screening, 529 (1.2%) contained T6SS genes, largely within Pseudomonadota, with sporadic occurrences in Bacteroidota, Cyanobacteriota, and Actinomycetota (Supplementary Table S5). Non-Pseudomonadota plasmids typically encoded partial clusters composed of *tssI/vgrG, tssE*, and occasionally *tssD/hcp* or *evpJ/PAAR*. Chromosomal T6SSs were likewise concentrated in Pseudomonadota (10 945 genomes), followed by Bacteroidota and a few additional phyla (Supplementary Table S6). Although this mirrors RefSeq taxonomic composition, it indicates that T6SSs are broadly distributed and not restricted to chromosomal loci in Pseudomonadota.

There is no universally accepted criterion for defining complete T6SS clusters in comparative genomic analyses. Previous studies analyzing broad genomic datasets have therefore used different thresholds depending on the dataset and detection strategy (e.g. ≥6 or ≥11 components [15, 50]). In this study, we defined putatively complete T6SSs as clusters containing at least eight genes. This threshold was derived from the bimodal distribution of T6SS component counts observed in our dataset (Fig. 1B), in which the second mode began at eight genes. Clusters meeting this threshold typically included proteins corresponding to all major structural modules of the T6SS. Because bioinformatic identification depends on sequence similarity thresholds, some components may remain undetected or may be genuinely absent. Applying this criterion, we identified 405 complete systems across 375 plasmids, with some plasmids carrying up to three T6SSs (Supplementary Table S7). Nearly all belonged to Pseudomonadota (n = 371), except for two cases in Bacteroidota and single representatives in Acidobacteriota and Gemmatimonadota. Among Pseudomonadota, plasmid-encoded T6SS^i^ (n = 403) were distributed across 20 host families, predominantly *Burkholderiaceae* (n = 138), *Rhizobiaceae* (n = 75), *Enterobacteriaceae* (n = 55), *Campylobacteraceae* (n = 19), *Rhodobacteraceae* (n = 14), *Vibrionaceae* (n = 13), and *Yersiniaceae* (n = 13). Two plasmid-encoded T6SS^iii^ were identified in Bacteroidota (NZ_CP027233.1 and NZ_CP095063.1). Chromosomes carried 14 789, 148, and 293 clusters of T6SS^i^, T6SS^ii^, and T6SS^iii^, respectively, some genomes encoding up to six T6SSs (Supplementary Table S8). A few genomes harbored more than one subtype: e.g. *Francisella* (four genomes) and *Dongshaea* (one genome) carried both T6SS^i^ and T6SS^ii^.

In addition to complete systems, clusters containing only two or three T6SS components and located outside canonical T6SS loci were frequently detected. These small regions typically included combinations of *tssD/hcp* and spike components (*tssI/vgrG* and/or *evpJ/PAAR*) (Fig. 1B). We identified 467 such loci on 239 plasmids and 7682 on 4806 chromosomes. Of the 239 plasmids, 109 lacked a complete T6SS but carried one to three of these smaller loci. Proportionally, these reduced genomic arrays were more prevalent in plasmids than in chromosomes (54% vs. 34%; Fisher's test, P = 6.97 × 10^−36^), indicating greater modularization of T6SS elements in mobile replicons.

These loci correspond to the so-called orphan islands [51, 52], defined as genomic regions that typically encode Hcp and/or spike-associated components and are not part of the canonical T6SS gene cluster [53]. Orphan islands frequently include adjacent effector and immunity gene pairs, and are therefore thought to expand the effector repertoire of resident T6SSs by providing interchangeable toxin delivery modules rather than encoding an autonomous secretion apparatus [54, 55]. Their presence suggests functional coupling to a complete T6SS encoded elsewhere in the genome and highlights their potential role in effector diversification and horizontal exchange. To further assess this, we searched for isolated individual *hcp* and *vgrG* genes, identifying 299 plasmids (183 with *vgrG* and 116 with *hcp*). Among them, 163 lacked both complete T6SSs and larger orphan islands. Altogether, 420 plasmids carried orphan islands (*hcp, vgrG, hcp-hcp, vgrG-vgrG, hcp-vgrG, hcp-PAAR, vgrG-PAAR, hcp-vgrG-PAAR*), of which 272 encoded only these islands without an associated complete T6SS (Supplementary Table S7).

### Plasmid genome size correlates with T6SS presence and complexity

**Figure 2:**
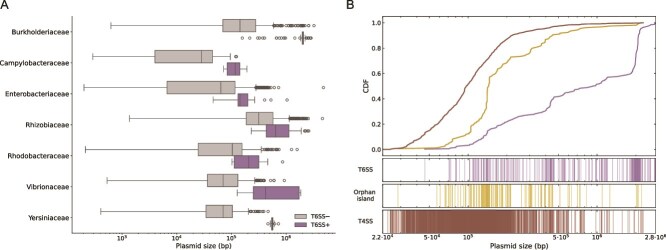
Genome size distribution of plasmids encoding T6SSs. (A) Distribution of plasmid sizes for the most abundant bacterial families, differentiated by the presence (purple) or absence (gray) of a complete T6SS. Statistical differences were assessed using a one-sided Mann–Whitney U test for each family: *Burholderiaceae* (*P =* 1.61 × 10^−61^), *Campylobacteraceae* (*P =* 5.26 × 10^−13^), *Enterobacteriaceae* (*P =* 1.22 × 10^−17^), *Rhizobiaceae* (*P =* 1.15 × 10^−13^), *Rhodobacteraceae* (*P =* 2.15 × 10^−5^), *Vibrionaceae* (*P =* 3.75 × 10^−8^) and *Yersiniaceae* (*P =* 3.52 × 10^−10^). (B) Distribution of T6SSs, orphan islands, and T4SSs in bacterial plasmids. A total of 3132 plasmids were ranked according to size (Supplementary Table S9). The top panel shows the CDF of genome size on a logarithmic scale for each plasmid subgroup. The bottom three panels display the presence of a complete T6SS (purple), orphan island (yellow) and T4SS (brown) for each genome. In all panels, the *x* axis represents size (bp) on log scale.

Natural plasmids span a broad size range across bacterial phyla, often varying by three orders of magnitude [22]. Across all bacterial families, plasmids encoding complete T6SSs clustered toward the upper end of their size distributions and were consistently larger than T6SS-negative counterparts (Fig. 2A). To contextualize this, we compared plasmid and host genome sizes. Using a ≥5% genome-size threshold to define megaplasmids [28] (Supplementary Table S3), 78.4% (294/375) of plasmids carrying complete T6SSs and 25.4% (69/272) plasmids with orphan islands met this criterion (Supplementary Table S7). Moreover, cumulative distribution analysis showed that plasmids encoding complete T6SSs (n = 375) were significantly larger than those carrying only T6SS orphan islands (n = 272; Mann–Whitney U test, *P =* 2.63 × 10^−43^; Fig. 2B).

The T6SS operon typically comprises 13–14 genes spanning >20 kb on average [56, 57], although some clusters are considerably larger [52]. While this substantial size could favor its retention on large plasmids, we tested whether operon length alone explains this association by comparing it to another multigene secretion system, the type IV secretion system (T4SS), which contains a comparable number of genes (~11 in the simplest MPF_T_ variant [58]). This comparison was used only as a size-comparable reference for a multigene locus. The cumulative size distribution of plasmids with T6SS was significantly shifted toward larger sizes relative to plasmids with T4SS (Mann–Whitney U test, *P =* 1.14 × 10^−131^; Fig. 2B), and only 6% of the latter group met the megaplasmid threshold (Supplementary Table S9), indicating that operon size alone cannot account for the strong link between T6SS presence and large plasmid size. Although T4SS distribution may itself reflect additional selective constraints across plasmid types, these results suggest that factors beyond locus size, such as energy cost, stability, or selective advantage, may contribute to the preferential maintenance of T6SSs on large plasmids.

### Genomic characteristics of T6SS-encoding plasmids

To determine whether distinctive genomic features of large T6SS-encoding plasmids influence bacterial ecology and evolution, we analyzed plasmids carrying either complete T6SS clusters or orphan islands. We evaluated their potential for horizontal transfer by screening for conjugation-associated modules, their maintenance strategies via partition and TA systems, and their accessory gene content, including AMR genes and virulence factors (see details in Materials and Methods).

**Figure 3:**
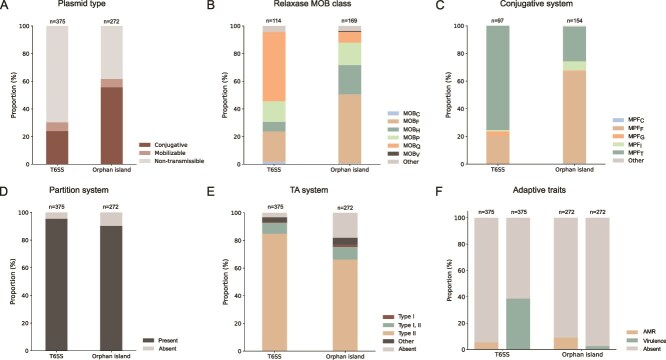
Mobility and functional trait associations of plasmids encoding complete T6SSs or orphan islands. (A) Proportion of conjugative, mobilizable and non-transmissible plasmids encoding a complete T6SS (n = 375) or an orphan island (n = 272), colored according to the legend. Proportion of plasmids encoding a complete T6SS or an orphan island according to (B) the MOB relaxase class, (C) the MPF type, (D) partition system, (E) TA module, and (F) AMR and virulence traits.

Plasmids were classified as transmissible (conjugative when both relaxase and mating-pair formation systems were present, or mobilizable when only relaxase was detected) or non-transmissible (lacking both). Plasmids carrying only orphan islands were highly enriched in transmissible categories compared with all plasmids carrying T6SS (62.1%, 169/272; 6.3% mobilizable, 55.8% conjugative; Fisher's test, *P =* 6.61 × 10^−16^), whereas a smaller fraction of those with complete T6SS clusters were transmissible (30.4%, 114/375; 6.4% mobilizable, 24% conjugative) (Fig. 3A; Supplementary Table S7).

When plasmids were subdivided according to both transmissibility and T6SS content, clear differences in GC divergence from host chromosomes were observed. Among transmissible plasmids, those encoding complete T6SS clusters showed a median GC content 3.507% lower than that of their hosts, whereas transmissible plasmids carrying orphan islands displayed a larger median difference (4.197%; Mann–Whitney U test, *P =* 6.66 × 10^−3^; Supplementary Fig. S1). In contrast, among non-transmissible plasmids, T6SS-encoding plasmids exhibited GC contents nearly identical to those of their host chromosomes (median difference 0.032%), while plasmids carrying orphan islands retained a larger divergence (median difference 1.542%; Mann–Whitney U test, *P =* 8.41 × 10^−12^; Supplementary Fig. S1).

Relaxase and conjugation system distributions were also different for plasmids encoding T6SS or orphan islands. MOB_F_ relaxases were most frequent (n = 109), especially among orphan island plasmids (n = 84; Fisher's test, *P =* 2.06 × 10^−6^), followed by MOB_Q_ (n = 70), mainly linked to complete T6SSs (n = 57; Fisher's test, *P =* 2.06 × 10^−6^) (Fig. 3B; Supplementary Table S7). MOB_P_ (n = 45) and MOB_H_ (n = 43) occurred less often, the latter mainly associated with plasmids encoding orphan islands (n = 35; Fisher's test, *P =* .002). Among mating-pair formation systems, MPF_F_ and MPF_T_ were predominant (122 and 112 plasmids, respectively): orphan-island plasmids were mainly MPF_F_-associated (n = 102; Fisher's test, *P =* 9.85 × 10^−13^), whereas complete T6SS plasmids preferentially carried MPF_T_ (n = 73; Fisher's test, *P =* 8.04 × 10^−15^) (Fig. 3C; Supplementary Table S7).

Partition systems were widespread, supporting stable inheritance of T6SS plasmids regardless of mobility potential. Most plasmids encoding complete clusters (358/375) and orphan islands (245/272) carried at least one partition protein, predominantly type I (ParA-ParB-like) (Fig. 3D, Supplementary Table S7). TA modules, especially type II, were also widespread both in plasmids carrying complete T6SS (363/375, median = 7) and orphan islands (223/272, median = 2) (Fig. 3E, Supplementary Table S7).

No significant association was observed between the presence of T6SS and AMR genes in plasmids. Only 45 encoded AMR determinants: 20 carrying complete T6SS and 25 with orphan islands (Fig. 3F, Supplementary Table S7). Although most T6SS-encoding plasmids lacked AMR genes, those with orphan islands contained significantly more AMR determinants (median = 8) than plasmids encoding complete T6SS clusters (median = 1; Mann–Whitney U test, *P =* 2.57 × 10^−9^) (Supplementary Table S7). Beyond AMR, we next examined the association between T6SS-encoding plasmids and virulence factors. These were more commonly associated with plasmids harboring complete T6SS clusters (145 of 375 plasmids) than with plasmids harboring orphan islands (7 of 272 plasmids; Fisher's test, *P =* 1.28 × 10^−29^) (Fig. 3F). The detected virulence genes were primarily associated with exotoxins and adherence mechanisms: *flgG* (n = 89), *fliI* (n = 87), *flgI* (n = 77), *cheW* (n = 30), *fliP* (n = 28) and *cheR* (n = 25) (Supplementary Table S7).

The ANI network of 375 T6SS-positive plasmids (Supplementary Fig. S2) revealed both densely connected groups and numerous singletons, reflecting high diversity and lineage-specific enrichment. Among these, 198 plasmids grouped into 13 PTUs across Pseudomonadota (Supplementary Table S7), with 10 PTUs showing host range grade II, defined as circulation among multiple species within a single genus (Supplementary Fig. S3). Each PTU exhibited a characteristic syntenic organization of its T6SS gene cluster (Supplementary Fig. S4). In contrast, 84 of the 272 plasmids carrying orphan islands were assigned to 26 PTUs across Pseudomonadota, encompassing host range grades I-IV (Supplementary Fig. S3). This pattern indicates a broader taxonomic distribution of orphan islands, extending up to families within a given order, compared with complete T6SS clusters.

T6SS-positive plasmids were enriched in metabolic functions (COG G, I, E), often intersecting with chromosomal pathways, as well as in inorganic ion transport (P) and occasionally energy production (C) (Supplementary Fig. S5), resembling megaplasmid profiles [59]. Analyses restricted to megaplasmids with or without T6SSs showed significant enrichments (Fisher’s exact test with Benjamini-Hochberg correction, adjusted *P <* .05) among T6SS-positive megaplasmids for signal transduction (T) in *Vibrionaceae* and *Rhodobacteraceae*, and amino acid metabolism (E) in *Rhizobiaceae*, suggesting an association between T6SS carriage and lineage-specific functional repertoires (Supplementary Fig. S5).

### Ecological and evolutionary analysis of T6SS-encoding PTUs

Horizontal gene transfer via plasmids offers bacteria a rapid route to acquire competitive traits such as the T6SS, a contact-dependent nanoweapon shaping microbial competition. However, the ecological significance of T6SS carriage at the level of persistent plasmid lineages (i.e. PTUs) remains underexplored. Here, we resolve relationships at the PTU level to assess whether specific plasmid lineages act as stable reservoirs, transient vectors, or evolutionary innovators of T6SSs.

**Figure 4:**
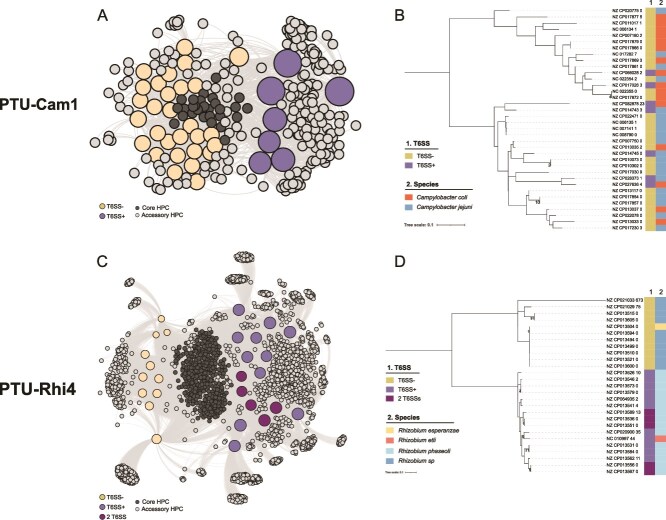
Features of PTU-Cam1 and PTU-Rhi4. (A) Bipartite proteome network of PTU-Cam1 performed with AcCNET at 80% identity and 80% coverage. Large nodes represent plasmids, colored according to the presence of T6SS. Smaller nodes depict homologous protein clusters (HPCs), colored dark gray if they are members of the PTU core proteome (present in ≥80% of PTU members) and light grey if they are not. (B) Core-genome phylogenetic tree of PTU-Cam1. Parsimony tree based on core SNPs. Branch length scale represents changes per number of SNPs. Numbers at the internal nodes indicate the SNPs shared exclusively by their descendant lineages. The tree was midpoint-rooted. Bars to the right of the tree indicate (i) T6SS presence and (ii) host species. (C) Bipartite proteome network of PTU-Rhi4, obtained and visualized as described for PTU-Cam1. (D) Core-genome phylogenetic tree of PTU-Rhi4, inferred and displayed as described for PTU-Cam1.

We constructed PTU-level bipartite networks, in which nodes represent plasmids and homologous protein clusters (HPCs) (Supplementary Fig. S6). Edges connect plasmids with HPCs whenever the latter contain a protein belonging to the plasmid. This network revealed whether T6SSs formed part of a PTU core proteome (≥80% of members), were sporadically present, or associated with distinct ecological subgroups. Several PTUs, including PTU-Bur3, PTU-E78, PTU-E79, and PTU-Rhi11, carried T6SSs as core features, indicating long-term stability. Others, such as PTU-Rhi3 and PTU-Rhi13, contained only a single T6SS-bearing member, consistent with sporadic acquisition, whereas PTU-Cam1 and PTU-Rhi4 exhibited intermediate patterns with T6SS-positive plasmids forming distinct subgroups, suggesting lineage-specific adaptation or niche specialization (Fig. 4). These subgroups illustrate how plasmid-encoded T6SSs contribute to diversification. To explore these dynamics, we integrated PTU-specific core genome phylogenies with pan-genome-wide association analyses (pan-GWAS) to identify accessory traits linked to T6SS carriage.

PTU-Cam1 represents a genus-restricted lineage of 36 *Campylobacter* plasmids (16 *C. coli*, 20 *C. jejuni*), of which seven encode complete T6SSs (4 *C. coli*, 3 *C. jejuni*). Network analysis (Fig. 4A) revealed a clear division between T6SS-positive and -negative subgroups, the former clustering cohesively through shared HPCs. T6SS-positive plasmids were significantly larger (median 138 kb) than T6SS-negative members (median 46 kb) (Mann–Whitney U test, *P =* 2.7 × 10^−5^), qualifying as megaplasmids (≥5% of the *Campylobacteraceae* genome size). Transposases of the IS*200*/IS*605* family, found exclusively near T6SS loci, may reflect occasional acquisition events involving these regions. Two conjugative megaplasmids previously shown to mediate hemolytic activity via T6SS (NZ_CP014745, NZ_CP014743; [17]) belong to this PTU, suggesting functional systems. Phylogeny shows T6SS presence is not the primary diversification driver (Fig. 4B). Nonetheless, pan-GWAS identified 99 genes associated with T6SS-positive plasmids, including uncharacterized loci, calcium/calmodulin-responsive adenylate cyclases, TA systems, and phage integrases (Supplementary Table S10). PTU-Cam1 thus exemplifies a stable plasmid lineage in which the T6SS has become an integrated feature, reflecting co-adaptation with *Campylobacter* hosts and likely reinforcing intraspecific competition and influencing pathogenic interactions.

PTU-Rhi4 comprises 27 *Rhizobium* plasmids (15 *R. phaseoli*, 10 *Rhizobium* sp., one each *R. esperanzae* and *R. etli*), 16 of which carry a T6SS; five encode two distinct systems. In this PTU, T6SS carriage correlates with ecological divergence within the genus *Rhizobium*, as T6SS-positive plasmids are restricted to *R. etli* and *R. phaseoli*, whereas T6SS-negative plasmids occur in other *Rhizobium* species. As in PTU-Cam1, T6SSs are not part of the core proteome (Fig. 4C), but T6SS-positive plasmids cluster together in the core-genome phylogeny, forming a distinct clade (Fig. 4D), reflecting intra-PTU diversification structured at the intra-genus level. The two T6SS variants differ in distribution: one is shared with PTU-Rhi3 and several unassigned *Rhizobium* plasmids, while the other is shared with PTU-Rhi6 and PTU-Rhi13 (Supplementary Fig. S4); both variants are confined to *Rhizobium*, suggesting variant-specific ecological roles. T6SS-positive plasmids are significantly larger (median 1.1 Mb) than T6SS-negative ones (median 751 kb; Mann–Whitney U test, *P =* 7.76 × 10^−6^) suggesting expansion of accessory content. Pan-GWAS identified enrichment for genes involved in inorganic ion transport/metabolism (COG P) and intracellular trafficking/secretion (COG U) (Supplementary Table S11), consistent with roles in competition or symbiosis in the plant rhizosphere. Taken together, these observations indicate that T6SS acquisition in PTU-Rhi4 is associated with ecological differentiation among *Rhizobium* species, likely reflecting adaptation to micro-niches shaped by resource partitioning.

### Phylogenetic relationships between chromosomal and plasmid-encoded T6SSs

**Figure 5:**
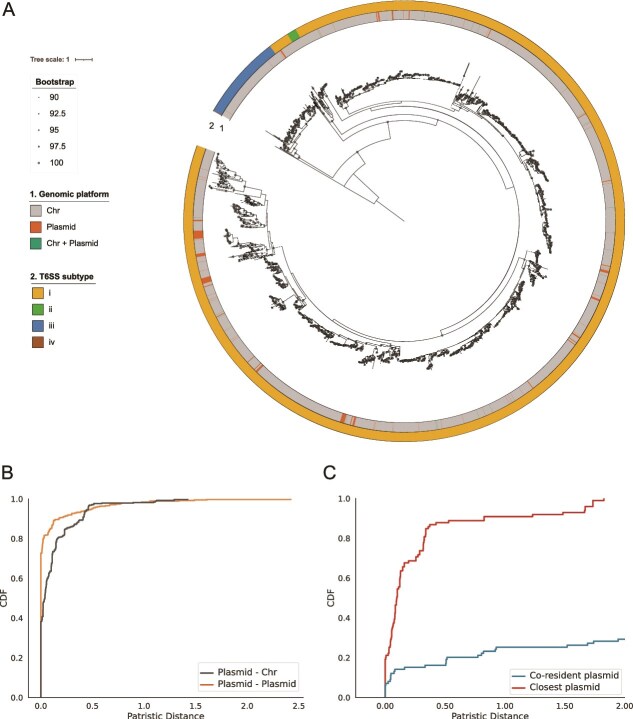
Phylogenetic analysis of T6SS. (A) Maximum-likelihood tree of the TssC homologs retrieved from T6SSs comprising ≥10 different components. The tree is rooted with the TssC homolog gp18, the T4 phage sheath subunit. Nodes with UF-Bootstrap support values ≥90% are indicated by gray circles. Rings, from inside to outside, indicate: (i) replicon (plasmid or chromosome) and (ii) T6SS subtype. (B and C) Cumulative distribution function of the patristic distances in the TssC phylogenetic tree. The curves represent the cumulative sum of the branch lengths linking each TssC to its closest homolog in the tree. (B) For each plasmid-encoded TssC subunit, the distance to its closest homolog in another plasmid (orange) and in a chromosome (gray) is shown. (C) For bacterial hosts encoding T6SS in both chromosome and plasmid (n = 62), the minimal patristic distance from the chromosomal TssC to its co-resident plasmid homolog (blue) and to its closest plasmid homolog (red). Statistical differences were evaluated using a one-sided Mann–Whitney U test (*P <* .001).

To assess plasmid-encoded T6SS evolution, we reconstructed a maximum-likelihood phylogeny of all non-redundant TssC subunits from clusters containing ≥10 components. We applied a stricter threshold of genes to retain clusters with a larger number of homologous components, thereby ensuring robust alignments and minimizing missing data. The resulting tree (Fig. 5A) recovered the expected deep clades (T6SS^iv^ (brown, *A. asiaticus* 5a2), Bacteroidetes-specific T6SS^iii^ (blue)) and the *Francisella*-associated T6SS^ii^ (green) nested within the broader T6SS^i^ lineage (yellow), confirming their evolutionary relationships. This analysis also revealed coexistence of T6SS^i^ and T6SS^ii^ systems within single genomes of *Francisella* and *Dongshaea*. Nearly all plasmid-encoded TssC subunits belonged to T6SS^i^, with only two clustering within T6SS^iii^.

Plasmid-derived TssCs were scattered throughout the phylogeny, frequently interspersed with chromosomal homologs (inner ring, Fig. 5A). Using ancestral state reconstruction [44], a phylogenetic approach that infers the likely presence or absence of traits in ancestral nodes, we traced the acquisition and transfer of T6SSs between plasmids and chromosomes across bacterial lineages. This approach indicated recurrent plasmid acquisition from chromosomal sources (Supplementary Fig. S7), followed by redistribution across diverse bacterial hosts (outer ring, Fig. 5A), underscoring plasmids as major vectors of T6SS dissemination. Near-identical TssC proteins (≥99% identity, 100% coverage) occurred on both plasmids and chromosomes, indicating recent exchange. The analysis also revealed multiple independent reintroductions of T6SSs from plasmids back into chromosomes (Supplementary Fig. S7), highlighting the bidirectional nature of these exchanges. Importantly, nearly identical copies often spanned different species, genera, and even families, demonstrating that plasmids can breach deep taxonomic barriers during T6SS transfer.

To compare the relatedness of plasmid and chromosomal T6SS subunits, we calculated patristic distances, reflecting the sum of branch lengths separating two nodes in the phylogeny and plotted their CDF, which represents the fraction of protein pairs with distances below each value in the tree. This visualization shows how closely proteins are related: a rapid initial increase indicates many closely related pairs, whereas slower rises reflect more divergent relationships. The CDF of patristic distances (Fig. 5B) showed a steep initial rise for both plasmid-plasmid and plasmid-chromosome comparisons, with the former dominating. Of 385 plasmid-encoded TssCs, 70.1% (n = 270) had identical homologs on another plasmid, consistent with tight clustering. Over half of these (53%; n = 143) also had near-identical chromosomal homologs, indicating frequent exchange between compartments. Only 3.6% (n = 14) matched exclusively to chromosomes. Even among non-identical pairs, plasmid-chromosome distances were short (median 0.11), suggesting ongoing transfers.

We next examined 62 isolates carrying T6SSs on both chromosomes and plasmids. For each, we compared the patristic distance between co-resident plasmid and chromosomal homologs to that between chromosomal TssCs and their closest plasmid homologs globally (Fig. 5C). Co-resident pairs were rarely the closest relatives (Mann–Whitney U test, *P =* 8.43 × 10^−20^), indicating dual systems arise from independent acquisitions rather than local duplications.

### Mechanisms of T6SS recruitment by plasmids

Phylogenetic analyses revealed extensive horizontal exchange of T6SSs between chromosomes and plasmids, with plasmid-plasmid transfers particularly frequent (Fig. 5A; Supplementary Fig. S7). To identify potential mechanisms, we examined the genetic context of T6SS loci for MGEs capable of mediating excision, mobilization, or integration, including phages, transposons, and insertion sequences (ISs). Specifically, we screened a 20-gene window around each chromosomal and plasmid-encoded T6SS for integrases, recombinases, and transposases.

Compared to chromosomes, plasmids showed a marked enrichment of MGE signatures near T6SSs or orphan islands (Fisher's test, *P =* 1.52 × 10^−60^). Among 910 orphan islands and 403 complete T6SS^i^ clusters, over half contained at least one adjacent MGE (Supplementary Fig. S8A). Members of the IS*5* and IS*3* families were the most common, associated with both complete T6SSs and orphan islands (Fisher's test, *P* > .05), whereas IS*110*-like elements were largely restricted to orphan islands (Fisher's test, *P =* 9.13 × 10^−10^) (Supplementary Fig. S8B). Chromosomal loci also harbored MGEs, but at lower frequencies: 3347 of 14 789 complete T6SS^i^ and 2863 of 7209 orphan islands contained nearby ISs (Supplementary Fig. S8A). On chromosomes, IS*3* (Fisher's test, *P =* 1.75 × 10^−5^) and IS*As1* members (Fisher's test, *P =* 1.23 × 10^−30^) were primarily linked to orphan islands, while IS*5* elements occurred more often near complete clusters (Fisher's test, *P =* 3.41 × 10^−28^) (Supplementary Fig. S8C).

### Recent transfer events between chromosomes and plasmids

**Figure 6:**
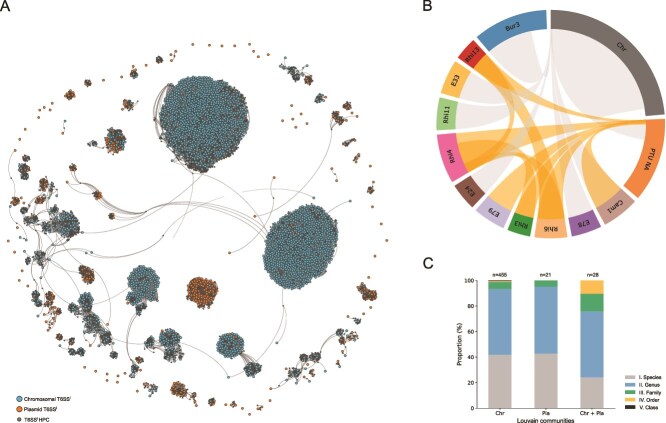
Recent horizontal gene transfer of T6SSs in plasmids and chromosomes. (A) Bipartite network of plasmid and chromosomal T6SSs^i^ generated at 99% identity and 100% coverage. Nodes representing T6SS proteins are shown as small gray circles, while nodes representing complete T6SSs are larger and color-coded by replicon: plasmid (orange) and chromosome (blue). Only Louvain communities composed of plasmid T6SSs or mixtures of plasmid and chromosomal T6SSs are shown. (B) Chord diagram showing connections in the network among PTUs, other plasmids (PTU NA), and chromosomes (Chr). Two genomic entities are linked by an edge when they belong to the same Louvain community. (C) Host range (I-V) of shared T6SS clusters detected within Louvain communities (I: when present in different strains of the same species; II: when present in different species of the same genus; III: when present in different genera of the same family; IV: when present in different families of the same order; V: when present in different orders of the same taxonomic class).

To assess recent T6SS transfers between plasmids and chromosomes, we constructed bipartite networks composed of two types of nodes: one representing replicons (plasmids or chromosomes) carrying a T6SS, and the other representing T6SS proteins. An edge connects a replicon to a protein when that protein is encoded on that replicon. Replicons sharing multiple T6SS proteins therefore become connected through common nodes, causing highly similar T6SS clusters to group together within the network and highlighting potential recent transfer events. The analysis focused on plasmid- and chromosome-encoded T6SS^i^ systems containing ≥10 distinct components and included 342 plasmid-encoded and 12 792 chromosomal systems. Proteins were clustered at 99% identity and 100% coverage to identify near-identical homologs indicative of recent horizontal transfer. In the resulting network (Fig. 6A), densely interconnected communities therefore highlight potential recent transfer events.

Community detection yielded 1256 clusters (Supplementary Table S12). Over half (n = 752) were singletons, including 57 plasmid-encoded systems, primarily located on plasmids outside defined PTUs. Among the 504 multi-member communities, most comprised closely related systems (Supplementary Fig. S9). Twenty-one communities contained only plasmid-encoded systems (n = 129), and several joined plasmids from distinct PTUs (Fig. 6B). For example, PTU-Rhi4 plasmids often carried two T6SSs. One clustered with PTU-Rhi3, whereas the other resembled PTU-Rhi6 or PTU-Rhi13, indicating inter-PTU recombination or capture from shared chromosomal sources.

A total of 156 plasmid-encoded T6SSs grouped with chromosomal counterparts (Supplementary Table S12), mainly within PTU-Bur3, PTU-Cam1, PTU-E33, PTU-Rhi11, PTU-E78, and PTU-E24 (Fig. 6B). Although chromosomal systems generally dominated these mixed communities, some (e.g. PTU-Cam1, PTU-Rhi11, PTU-E24) contained comparable plasmid and chromosomal representation. Of the 28 Louvain communities that included T6SSs on both chromosomes and plasmids, nine showed a transposase of the same IS family located near the system on both replicon types. This pattern occurred, e.g. in mixed communities that included T6SSs on PTU-E33 or PTU-E78 plasmids and chromosomes. Most transfers occurred between closely related taxa, typically within species or genera, with a minority of mixed communities spanning distinct families (Fig. 6C).

## Discussion

Our comparative genomic analyses reveal that T6SSs are not confined to chromosomal loci but are also encoded on plasmids, expanding current views of how machineries mediating interbacterial antagonism are organized and propagated across bacterial genomes. Co-occurring T6SS systems differing between plasmids and chromosomes of the same host suggest that plasmid localization broadens functional repertoires rather than merely increasing gene dosage, mirroring diversification among co-resident chromosomal T6SS loci. We found that plasmid-encoded T6SSs are widespread across Pseudomonadota families but display a marked bias toward megaplasmids, suggesting that these replicons provide suitable contexts for maintaining complex machineries.

Beyond their size, megaplasmids are characterized by chromosome-like features that stabilize low-copy replicons. In *Enterobacteriaceae* large plasmids encode ParAB partition systems and FtsK-dependent dimer resolution modules that safeguard faithful inheritance [60, 61]. These mechanisms ensure correct segregation and tether plasmids to the nucleoid core [61]. Large plasmids also frequently encode TA systems, which contribute to plasmid stability through post-segregational killing mechanisms [62]. In this context, the association observed between plasmid-encoded T6SS clusters and TA systems may reflect the contribution of plasmid stability mechanisms to the maintenance of energetically costly loci such as T6SS, although our data do not allow us to determine whether TA systems are required for T6SS carriage or simply co-occur with it. Scaling laws in plasmid biology show that larger plasmids exhibit lower copy numbers and converge toward chromosomal organization [63], carrying tRNAs [64, 65], transcription- and translation-related genes [66], and nucleoid-associated proteins such as H-NS [67, 68]. Consequently, the strong association of complete T6SSs with megaplasmids likely reflects the capacity of these replicons to integrate, maintain, and disseminate complex adaptive traits.

The ecological consequences of T6SS carriage are context dependent. Some studies with chromosomal T6SSs report little or no fitness cost under laboratory conditions [69, 70], whereas others reveal strong counter-selection in host-like or symbiotic environments [71–73]. This suggests that the benefits of megaplasmid-encoded T6SS may be conditional, providing advantages in competitive or specific environmental contexts. Accordingly, energy cost, stability requirements, or selective advantages in particular environments, may drive the preferential maintenance of complete T6SSs on large plasmids.

We found that plasmids carrying orphan islands were more frequently associated with conjugative systems than those encoding complete T6SS loci (Fig. 3), indicating that modular, T6SS-related cargo is particularly amenable to horizontal transfer. Consistently, plasmids with orphan islands exhibited broader host ranges than those carrying full T6SSs (Supplementary Fig. S3). These findings indicate that plasmids mobilize individual *hcp* and *vgrG* modules independently of the full secretion apparatus, potentially facilitating the modular exchange of effector genes. These orphan islands therefore act as flexible genomic platforms enabling rapid turnover of toxic effectors, promoting functional diversification without requiring mobilization of the entire secretion apparatus. Different conjugation machineries are associated with T6SS and orphan islands, potentially reflecting differences in mobilization dynamics. However, whether these associations instead reflect selective compatibility between plasmid backbones and specific cargo, or differential long-term maintenance of costly loci such as complete T6SS clusters, remains to be determined.

Within PTUs, T6SS distribution is highly heterogeneous. Some plasmid lineages retain T6SSs as stable, core features, whereas others exhibit sporadic or lineage-restricted occurrences. This diversity suggests multiple evolutionary trajectories, from long-term integration to transient acquisition. Certain families appear to have domesticated T6SSs, while others behave as transient vectors. Thus, plasmids can function both as long-term repositories and transient carriers of antagonistic potential, reflecting distinct evolutionary trajectories across plasmid lineages.

Genetic elements recently acquired by horizontal transfer frequently exhibit atypical nucleotide composition and gradually converge toward that of the host genome through amelioration processes affecting GC content and other sequence signatures [59, 74, 75]. In this context, the near-identical GC content between non-transmissible T6SS-encoding plasmids and their host chromosomes, contrasted with the larger divergence observed for transmissible T6SS plasmids, is consistent with a longer-term association between these plasmids and their hosts. These patterns suggest that horizontal transfer may preferentially disseminate effector modules, whereas complete T6SS machineries tend to be stably maintained within specific plasmid-host contexts.

The mobilization of complete T6SS loci likely reflects physical and genetic constraints on horizontal gene transfer. Typical transfer tracts of 25–30 kb per transfer event [76, 77] suffice to capture a full T6SS locus, yet such events are favored in the context of large MGEs [78]. Our phylogenetic analyses (Fig. 5A and Supplementary Fig. S7) indicated repeated acquisition of plasmid-borne T6SSs from chromosomal origins. This observation broadens our understanding of T6SS biology, showing that plasmids offer a structural context that overcomes the size constraints that restrict the transfer of full T6SS loci through natural transformation, which generally favors the acquisition of only a few genes at a time [78, 79]. Nearly identical T6SSs detected across distinct plasmid lineages and chromosomes (Fig. 6), often flanked by transposases, offer direct evidence of ongoing bidirectional exchange across species, genera, and even taxonomic families.

The recurrent association of insertion sequences with T6SS loci underscores the potential role of mobile genetic elements in facilitating these exchanges. In particular, the enrichment and close proximity of IS*3*- and IS*5*-family elements to complete systems on plasmids suggest that these ISs may act as molecular “handles” enabling plasmids to capture and redistribute T6SS clusters. Conversely, their lower frequencies at chromosomal loci imply that chromosomes may function as reservoirs from which plasmids repeatedly acquire T6SS modules. These recurrent transfers highlight plasmids as active agents accelerating the redistribution of complex adaptive systems. Similar dynamics underlie the spread of toxin and antibiotic-resistance modules [80, 81], emphasizing that plasmids serve simultaneously as transient vehicles and enduring scaffolds for antagonistic innovations. Nevertheless, most T6SS-sharing communities remain phylogenetically constrained, suggesting that horizontal transfer generally occurs among closely related hosts, although occasional events may bridge deeper evolutionary divergences.

Plasmid-encoded T6SSs contribute to diverse ecological outcomes. In *Vibrio crassostreae* and *Rhizobium*, plasmid-borne T6SSs enhance host colonization or mutualistic symbiosis [16, 19, 82]. In commensal *Neisseria cinerea*, a plasmid-encoded T6SS enables outcompetition of related pathogens [18], while in *Campylobacter*, megaplasmid T6SSs enable erythrocyte lysis, facilitating persistence in blood-rich environments [17]. These examples illustrate that plasmid-encoded T6SSs can be antagonistic, symbiotic, or pathogenic tools depending on environmental context. In contrast, the preferential linkage of compact orphan islands to conjugative plasmids suggests a modular dissemination strategy, that spreads specific effectors without the full apparatus. This division of labor mirrors an evolutionary balance between stability and mobility: megaplasmids provide long-term innovation hubs, whereas smaller plasmids accelerate effector turnover.

Such mobility has substantial ecological implications: a subset of plasmids encoding either T6SS or orphan islands in our dataset are conjugative (Fig. 3), providing a mechanism for the rapid introduction of antagonistic or virulence traits into novel hosts and reshaping microbial interactions across environments, from structuring competitive dynamics in microbiomes to accelerating the emergence of clinical pathogens with expanded virulence repertoires.

Collectively, our findings support a mechanistic and ecological model in which plasmids act as both reservoirs and vectors of T6SSs, shaping bacterial competitiveness and adaptation across environments. The preferential association of complete T6SSs with large, stably maintained plasmids underscores that these systems are not passive cargo but integrated components of plasmid-host consortia. By coupling stability with mobility, plasmids enable the continuous recruitment, refinement, and redeployment of antagonistic modules, influencing microbial community structure and evolutionary trajectories. Future work should explore how these dynamics affect community resilience, cooperation-competition balance, and the long-term diversification of microbial lineages. While our study is based on comparative genomic analyses and therefore primarily identifies correlations between plasmid size, mobility traits, and T6SS content, our data provide genome-wide support for the evolutionary dynamics and potential functional specialization of plasmid-borne T6SSs. Nevertheless, as these inferences are correlative, experimental validation will be necessary to confirm causality and fully understand the functional consequences of these genomic trends.

## Supplementary Material

ycag069_Supplemental_Files

## Data Availability

The authors confirm all supporting data and protocols have been provided either within the article, in supplementary data files, or through supporting data files and bioinformatic pipelines available on GitHub (https://github.com/mdmqc/Plasmid_T6SS).

## References

[1] Jamali H, Akrami F, Layeghkhavidaki H. et al. Bacterial protein secretion systems: mechanisms, functions, and roles in virulence. *Microb Pathog* 2025;206:107790. 10.1016/j.micpath.2025.107790 40480450

[2] Allsopp LP, Bernal P. Killing in the name of: T6SS structure and effector diversity. *Microbiology* 2023;169:001367. 10.1099/mic.0.001367 37490402 PMC10433429

[3] Jurėnas D, Journet L. Activity, delivery, and diversity of type VI secretion effectors. *Mol Microbiol* 2021;115:383–94. 10.1111/mmi.14648 33217073

[4] Cherrak Y, Flaugnatti N, Durand E. et al. Structure and activity of the type VI secretion system. *Microbiol Spectrum* 2019;7. 10.1128/microbiolspec.PSIB-0031-2019 PMC10957189 31298206

[5] Wood TE, Aksoy E, Hachani A. From welfare to warfare: the arbitration of host-microbiota interplay by the type VI secretion system. *Front Cell Infect Microbiol* 2020;10:587948. 10.3389/fcimb.2020.587948 33194832 PMC7604300

[6] Lin J, Xu L, Yang J. et al. Beyond dueling: roles of the type VI secretion system in microbiome modulation, pathogenesis and stress resistance. *Stress Biol* 2021;1:11. 10.1007/s44154-021-00008-z 37676535 PMC10441901

[7] Basler M, Pilhofer M, Henderson GP. et al. Type VI secretion requires a dynamic contractile phage tail-like structure. *Nature* 2012;483:182–6. 10.1038/nature10846 22367545 PMC3527127

[8] Leiman PG, Basler M, Ramagopal UA. et al. Type VI secretion apparatus and phage tail-associated protein complexes share a common evolutionary origin. *Proc Natl Acad Sci* 2009;106:4154–9. 10.1073/pnas.0813360106 19251641 PMC2657435

[9] Durand E, Nguyen VS, Zoued A. et al. Biogenesis and structure of a type VI secretion membrane core complex. *Nature* 2015;523:555–60. 10.1038/nature14667 26200339

[10] Nazarov S, Schneider JP, Brackmann M. et al. Cryo-EM reconstruction of type VI secretion system baseplate and sheath distal end. *EMBO J* 2018;37:e97103. 10.15252/embj.201797103 29255010 PMC5813253

[11] Bönemann G, Pietrosiuk A, Diemand A. et al. Remodelling of VipA/VipB tubules by ClpV-mediated threading is crucial for type VI protein secretion. *EMBO J* 2009;28:315–25. 10.1038/emboj.2008.269 19131969 PMC2646146

[12] Shneider MM, Buth SA, Ho BT. et al. PAAR-repeat proteins sharpen and diversify the type VI secretion system spike. *Nature* 2013;500:350–3. 10.1038/nature12453 23925114 PMC3792578

[13] Cianfanelli FR, Monlezun L, Coulthurst SJ. Aim, load, fire: the type VI secretion system, a bacterial Nanoweapon. *Trends Microbiol* 2016;24:51–62. 10.1016/j.tim.2015.10.005 26549582

[14] Unni R, Pintor KL, Diepold A. et al. Presence and absence of type VI secretion systems in bacteria. *Microbiology* 2022;168. 10.1099/mic.0.001151 35467500

[15] Morgado S, Vicente AC. Diversity and distribution of type VI secretion system gene clusters in bacterial plasmids. *Sci Rep* 2022;12:8249. 10.1038/s41598-022-12382-3 35581398 PMC9113992

[16] Piel D, Bruto M, James A. et al. Selection of vibrio crassostreae relies on a plasmid expressing a type 6 secretion system cytotoxic for host immune cells. *Environ Microbiol* 2020;22:4198–211. 10.1111/1462-2920.14776 31390475

[17] Marasini D, Karki AB, Bryant JM. et al. Molecular characterization of megaplasmids encoding the type VI secretion system in campylobacter jejuni isolated from chicken livers and gizzards. *Sci Rep* 2020;10:12514. 10.1038/s41598-020-69155-z 32719325 PMC7385129

[18] Custodio R, Ford RM, Ellison CJ. et al. Type VI secretion system killing by commensal Neisseria is influenced by expression of type four pili. *Elife* 2021;10:e63755. 10.7554/eLife.63755 34232858 PMC8263058

[19] Salinero-Lanzarote A, Pacheco-Moreno A, Domingo-Serrano L. et al. The type VI secretion system of rhizobium etli Mim1 has a positive effect in symbiosis. *FEMS Microbiol Ecol* 2019;95:fiz054. 10.1093/femsec/fiz054 30977796

[20] Redondo-Salvo S, Fernández-López R, Ruiz R. et al. Pathways for horizontal gene transfer in bacteria revealed by a global map of their plasmids. *Nat Commun* 2020;11:3602. 10.1038/s41467-020-17278-2 32681114 PMC7367871

[21] Rodríguez-Beltrán J, DelaFuente J, León-Sampedro R. et al. Beyond horizontal gene transfer: the role of plasmids in bacterial evolution. *Nat Rev Microbiol* 2021;19:347–59. 10.1038/s41579-020-00497-1 33469168

[22] Garcillán-Barcia MP, de la Cruz F, Rocha EPC. The extended mobility of plasmids. *Nucleic Acids Res* 2025;53:gkaf652. 10.1093/nar/gkaf652 40694848 PMC12282955

[23] Peñil-Celis A, Garcillán-Barcia MP. Crosstalk between type VI secretion system and Mobile genetic elements. *Front Mol Biosci* 2019;6:126. 10.3389/fmolb.2019.00126 31799257 PMC6863884

[24] Orlek A, Phan H, Sheppard AE. et al. Ordering the mob: insights into replicon and MOB typing schemes from analysis of a curated dataset of publicly available plasmids. *Plasmid* 2017;91:42–52. 10.1016/j.plasmid.2017.03.002 28286183 PMC5466382

[25] Abby SS, Néron B, Ménager H. et al. MacSyFinder: a program to mine genomes for molecular systems with an application to CRISPR-Cas systems. *PLoS One* 2014;9:e110726. 10.1371/journal.pone.0110726 25330359 PMC4201578

[26] Gilchrist CLM, Chooi Y-H. Clinker &amp; clustermap.Js: automatic generation of gene cluster comparison figures. *Bioinformatics* 2021;37:2473–5. 10.1093/bioinformatics/btab007 33459763

[27] Redondo-Salvo S, Bartomeus-Peñalver R, Vielva L. et al. COPLA, a taxonomic classifier of plasmids. *BMC Bioinformatics* 2021;22:390. 10.1186/s12859-021-04299-x 34332528 PMC8325299

[28] Hall JPJ, Botelho J, Cazares A. et al. What makes a megaplasmid? *Philos Trans R Soc B Biol Sci* 2022;377:20200472. 10.1098/rstb.2020.0472 PMC8628078 34839707

[29] Yoon S-H, Ha S-M, Kwon S. et al. Introducing EzBioCloud: a taxonomically united database of 16S rRNA gene sequences and whole-genome assemblies. *Int J Syst Evol Microbiol* 2017;67:1613–7. 10.1099/ijsem.0.001755 28005526 PMC5563544

[30] Garcillán-Barcia MP, Redondo-Salvo S, Vielva L. et al. MOBscan: Automated annotation of MOB Relaxases. In: de la Cruz F. (ed.), Horizontal Gene Transfer. Methods in Molecular Biology, Vol. 2075. New York, NY: Humana Press, 2020, 295–308. 10.1007/978-1-4939-9877-7_21 31584171

[31] Cury J, Abby SS, Doppelt-Azeroual O. et al. Identifying conjugative plasmids and integrative conjugative elements with CONJscan. In: de la Cruz F. (ed.), Horizontal Gene Transfer. Methods in Molecular Biology, Vol. 2075. New York, NY: Humana Press, 2020, 265–83. 10.1007/978-1-4939-9877-7_19 31584169

[32] Bharat A, Petkau A, Avery BP. et al. Correlation between phenotypic and In Silico detection of antimicrobial resistance in salmonella enterica in Canada using Staramr. *Microorganisms* 2022;10:292. 10.3390/microorganisms10020292 35208747 PMC8875511

[33] Liu B, Zheng D, Zhou S. et al. VFDB 2022: a general classification scheme for bacterial virulence factors. *Nucleic Acids Res* 2022;50:D912–7. 10.1093/nar/gkab1107 34850947 PMC8728188

[34] Siguier P . ISfinder: the reference Centre for bacterial insertion sequences. *Nucleic Acids Res* 2006;34:D32–6. 10.1093/nar/gkj014 16381877 PMC1347377

[35] Guan J, Chen Y, Goh Y-X. et al. TADB 3.0: an updated database of bacterial toxin–antitoxin loci and associated mobile genetic elements. *Nucleic Acids Res* 2024;52:D784–90. 10.1093/nar/gkad962 37897352 PMC10767807

[36] Steinegger M, Söding J. MMseqs2 enables sensitive protein sequence searching for the analysis of massive data sets. *Nat Biotechnol* 2017;35:1026–8. 10.1038/nbt.3988 29035372

[37] Katoh K, Standley DM. MAFFT multiple sequence alignment software version 7: improvements in performance and usability. *Mol Biol Evol* 2013;30:772–80. 10.1093/molbev/mst010 23329690 PMC3603318

[38] Capella-Gutiérrez S, Silla-Martínez JM, Gabaldón T. trimAl: a tool for automated alignment trimming in large-scale phylogenetic analyses. *Bioinformatics* 2009;25:1972–3. 10.1093/bioinformatics/btp348 19505945 PMC2712344

[39] Nguyen L-T, Schmidt HA, von Haeseler A. et al. IQ-TREE: a fast and effective stochastic algorithm for estimating maximum-likelihood phylogenies. *Mol Biol Evol* 2015;32:268–74. 10.1093/molbev/msu300 25371430 PMC4271533

[40] Kalyaanamoorthy S, Minh BQ, Wong TKF. et al. ModelFinder: fast model selection for accurate phylogenetic estimates. *Nat Methods* 2017;14:587–9. 10.1038/nmeth.4285 28481363 PMC5453245

[41] Minh BQ, Nguyen MAT, von Haeseler A. Ultrafast approximation for phylogenetic bootstrap. *Mol Biol Evol* 2013;30:1188–95. 10.1093/molbev/mst024 23418397 PMC3670741

[42] Gardner SN, Slezak T, Hall BG. kSNP3.0: SNP detection and phylogenetic analysis of genomes without genome alignment or reference genome. *Bioinformatics* 2015;31:2877–8. 10.1093/bioinformatics/btv271 25913206

[43] Letunic I, Bork P. Interactive tree of life (iTOL) v5: an online tool for phylogenetic tree display and annotation. *Nucleic Acids Res* 2021;49:W293–6. 10.1093/nar/gkab301 33885785 PMC8265157

[44] Ishikawa SA, Zhukova A, Iwasaki W. et al. A fast likelihood method to reconstruct and visualize ancestral scenarios. *Mol Biol Evol* 2019;36:2069–85. 10.1093/molbev/msz131 31127303 PMC6735705

[45] Lanza VF, Baquero F, de la Cruz F. et al. AcCNET (accessory genome constellation network): comparative genomics software for accessory genome analysis using bipartite networks. *Bioinformatics* 2017;33:283–5. 10.1093/bioinformatics/btw601 27663497

[46] Page AJ, Cummins CA, Hunt M. et al. Roary: rapid large-scale prokaryote pan genome analysis. *Bioinformatics* 2015;31:3691–3. 10.1093/bioinformatics/btv421 26198102 PMC4817141

[47] Brynildsrud O, Bohlin J, Scheffer L. et al. Rapid scoring of genes in microbial pan-genome-wide association studies with Scoary. *Genome Biol* 2016;17:238. 10.1186/s13059-016-1108-8 27887642 PMC5124306

[48] Huerta-Cepas J, Szklarczyk D, Heller D. et al. eggNOG 5.0: a hierarchical, functionally and phylogenetically annotated orthology resource based on 5090 organisms and 2502 viruses. *Nucleic Acids Res* 2019;47:D309–14. 10.1093/nar/gky1085 30418610 PMC6324079

[49] Cantalapiedra CP, Hernández-Plaza A, Letunic I. et al. eggNOG-mapper v2: functional annotation, Orthology assignments, and domain prediction at the metagenomic scale. *Mol Biol Evol* 2021;38:5825–9. 10.1093/molbev/msab293 34597405 PMC8662613

[50] Abby SS, Cury J, Guglielmini J. et al. Identification of protein secretion systems in bacterial genomes. *Sci Rep* 2016;6:23080. 10.1038/srep23080 26979785 PMC4793230

[51] Sarris PF, Skandalis N, Kokkinidis M. et al. In silico analysis reveals multiple putative type VI secretion systems and effector proteins in pseudomonas syringae pathovars. *Mol Plant Pathol* 2010;11:795–804. 10.1111/j.1364-3703.2010.00644.x 21091602 PMC6640432

[52] Blondel CJ, Jiménez JC, Contreras I. et al. Comparative genomic analysis uncovers 3 novel loci encoding type six secretion systems differentially distributed in salmonella serotypes. *BMC Genomics* 2009;10:354. 10.1186/1471-2164-10-354 19653904 PMC2907695

[53] Journet L, Cascales E. The type VI secretion system in Escherichia coli and related species. *EcoSal Plus* 2016;7. 10.1128/ecosalplus.esp-0009-2015 PMC11575709 27223818

[54] Barret M, Egan F, Fargier E. et al. Genomic analysis of the type VI secretion systems in pseudomonas spp.: novel clusters and putative effectors uncovered. *Microbiology* 2011;157:1726–39. 10.1099/mic.0.048645-0 21474537

[55] De Maayer P, Venter SN, Kamber T. et al. Comparative genomics of the type VI secretion systems of Pantoea and Erwinia species reveals the presence of putative effector islands that may be translocated by the VgrG and hcp proteins. *BMC Genomics* 2011;12:576. 10.1186/1471-2164-12-576 22115407 PMC3235180

[56] Silverman JM, Brunet YR, Cascales E. et al. Structure and regulation of the type VI secretion system. *Ann Rev Microbiol* 2012;66:453–72. 10.1146/annurev-micro-121809-151619 22746332 PMC3595004

[57] Boyer F, Fichant G, Berthod J. et al. Dissecting the bacterial type VI secretion system by a genome wide in silico analysis: what can be learned from available microbial genomic resources? *BMC Genomics* 2009;10:104. 10.1186/1471-2164-10-104 19284603 PMC2660368

[58] Guglielmini J, Néron B, Abby SS. et al. Key components of the eight classes of type IV secretion systems involved in bacterial conjugation or protein secretion. *Nucleic Acids Res* 2014;42:5715–27. 10.1093/nar/gku194 24623814 PMC4027160

[59] diCenzo GC, Finan TM. The divided bacterial genome: structure, function, and evolution. *Microbiol Mol Biol Rev* 2017;81:e00019-17. 10.1128/MMBR.00019-17 28794225 PMC5584315

[60] Fournes F, Campos M, Cury J. et al. The pathway to resolve dimeric forms distinguishes plasmids from megaplasmids in Enterobacteriaceae. *Nucleic Acids Res* 2025;53:gkae1300. 10.1093/nar/gkae1300 39797729 PMC11724359

[61] Planchenault C, Pons MC, Schiavon C. et al. Intracellular positioning systems limit the entropic eviction of secondary replicons toward the nucleoid edges in bacterial cells. *J Mol Biol* 2020;432:745–61. 10.1016/j.jmb.2019.11.027 31931015

[62] Effe J, Santer M, Wang Y. et al. The combination of active partitioning and toxin-antitoxin systems is most advantageous for low-copy plasmid fitness. *Nat Commun* 2025;16:7078. 10.1038/s41467-025-62473-8 40750619 PMC12317039

[63] Maddamsetti R, Shyti I, Wilson ML. et al. Scaling laws of bacterial and archaeal plasmids. *Nat Commun* 2025;16:6023. 10.1038/s41467-025-61205-2 40603865 PMC12222811

[64] diCenzo G, Milunovic B, Cheng J. et al. The tRNA arg gene and engA are essential genes on the 1.7-Mb pSymB Megaplasmid of Sinorhizobium meliloti and were translocated together from the chromosome in an ancestral strain. *J Bacteriol* 2013;195:202–12. 10.1128/JB.01758-12 23123907 PMC3553834

[65] Dineen RL, Bottacini F, O’Connell-Motherway M. et al. Transcriptional landscape of the pMP7017 megaplasmid and its impact on the Bifidobacterium breve UCC2003 transcriptome. *Microb Biotechnol* 2024;17:e14405. 10.1111/1751-7915.14405 38206097 PMC10832533

[66] Harrison PW, Lower RPJ, Kim NKD. et al. Introducing the bacterial ‘chromid’: not a chromosome, not a plasmid. *Trends Microbiol* 2010;18:141–8. 10.1016/j.tim.2009.12.010 20080407

[67] Fitzgerald S, Kary SC, Alshabib EY. et al. Redefining the H-NS protein family: a diversity of specialized core and accessory forms exhibit hierarchical transcriptional network integration. *Nucleic Acids Res* 2020;48:10184–98. 10.1093/nar/gkaa709 32894292 PMC7544231

[68] Takeda T, Yun C-S, Shintani M. et al. Distribution of genes encoding nucleoid-associated protein homologs in plasmids. *Int J Evol Biol* 2011;2011:1–30. 10.4061/2011/685015 PMC3042613 21350637

[69] Taillefer B, Giraud JF, Cascales E. No fitness cost entailed by type VI secretion system synthesis, assembly, contraction, or disassembly in enteroaggregative Escherichia coli. *J Bacteriol* 2023;205:e0035723. 10.1128/jb.00357-23 37971272 PMC10729742

[70] Zhang C, Datta S, Ratcliff WC. et al. Constitutive expression of the type VI secretion system carries no measurable fitness cost in vibrio cholerae. *Ecol Evol* 2024;14:e11081. 10.1002/ece3.11081 38435022 PMC10905242

[71] Robitaille S, Simmons EL, Verster AJ. et al. Community composition and the environment modulate the population dynamics of type VI secretion in human gut bacteria. *Nat Ecol Evol* 2023;7:2092–107. 10.1038/s41559-023-02230-6 37884689 PMC11099977

[72] Septer AN, Sharpe G, Shook EA. The Vibrio fischeri type VI secretion system incurs a fitness cost under host-like conditions. *bioRxiv (preprint)* 2023. 10.1101/2023.03.07.529561

[73] Gupta S, Ray S, Khan A. et al. The cost of bacterial predation via type VI secretion system leads to predator extinction under environmental stress. *iScience* 2021;24:103507. 10.1016/j.isci.2021.103507 34934926 PMC8654991

[74] Rocha EPC, Danchin A. Base composition bias might result from competition for metabolic resources. *Trends Genet* 2002;18:291–4. 10.1016/S0168-9525(02)02690-2 12044357

[75] Lawrence JG, Ochman H. Amelioration of bacterial genomes: rates of change and exchange. *J Mol Evol* 1997;44:383–97. 10.1007/PL00006158 9089078

[76] Pang TY, Lercher MJ. Supra-operonic clusters of functionally related genes (SOCs) are a source of horizontal gene co-transfers. *Sci Rep* 2017;7:40294. 10.1038/srep40294 28067311 PMC5220362

[77] Pang TY, Lercher MJ. Each of 3,323 metabolic innovations in the evolution of E. Coli arose through the horizontal transfer of a single DNA segment. *Proc Natl Acad Sci* 2019;116:187–92. 10.1073/pnas.1718997115 30563853 PMC6320504

[78] Mazzamurro F, Touchon M, Charpentier X. et al. Impact of natural transformation on the Acquisition of Novel Genes in bacteria. *Mol Biol Evol* 2025;42:msaf192. 10.1093/molbev/msaf192 40794765 PMC12359135

[79] Power JJ, Pinheiro F, Pompei S. et al. Adaptive evolution of hybrid bacteria by horizontal gene transfer. *Proc Natl Acad Sci* 2021;118:e2007873118. 10.1073/pnas.2007873118 33649202 PMC7958396

[80] Coluzzi C, Rocha EPC. The spread of antibiotic resistance is driven by plasmids among the fastest evolving and of broadest host range. *Mol Biol Evol* 2025;42:msaf060. 10.1093/molbev/msaf060 40098486 PMC11952959

[81] Shapiro JT, Zorea A, Kav AB. et al. Multilayer networks of plasmid genetic similarity reveal potential pathways of gene transmission. *ISME J* 2023;17:649–59. 10.1038/s41396-023-01373-5 36759552 PMC10119158

[82] De Sousa BFS, Domingo-Serrano L, Salinero-Lanzarote A. et al. The T6SS-dependent effector Re78 of rhizobium etli Mim1 benefits bacterial competition. *Biology (Basel)* 2023;12:678. 10.3390/biology12050678 37237492 PMC10215855

