# Supplementary material for: Plasmids serve as vehicles and reservoirs of type VI secretion systems
Source: ISME Commun. 2026 Mar 23;6(1):ycag069. doi: 10.1093/ismeco/ycag069 (PMC13098142; doi:10.1093/ismeco/ycag069)
Supplement: ycag069_Supplemental_Files [file ycag069_supplemental_files.zip › renamed_6f9c2_ycag069.pdf]

A

	Non-transmissible		Transmissible	
	Pla – Chr comparisons	Median GC (%)	Pla – Chr comparisons	Median GC (%)
None	16,014	-2.802	15,671	-2.933
Orphan island	113	-1.542	171	-4.197
T6SS	257	-0.032	113	-3.507

B

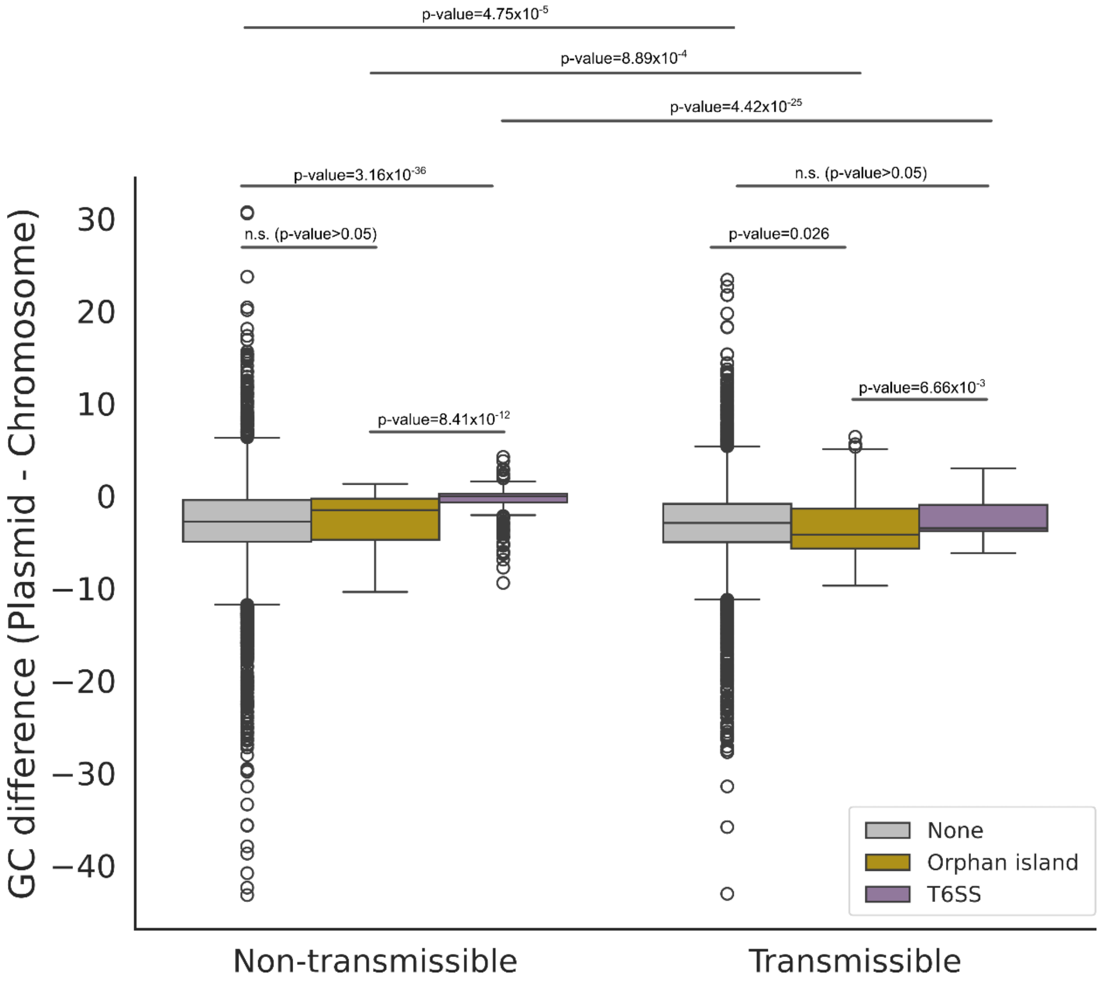

Supplementary Figure S1. Comparison of GC content between co-resident plasmids and chromosomes. A) Number of plasmid-chromosome pairs used for the analysis of GC content. Along with median GC values for each group. B) Boxplots showing the difference in GC content between plasmids and their host chromosomes stratified by plasmid mobility (non-transmissible or transmissible by conjugation) and T6SS type (putative complete T6SS, orphan islands, or none). P-values derived from the one-sided Mann-Whitney U tests are shown.

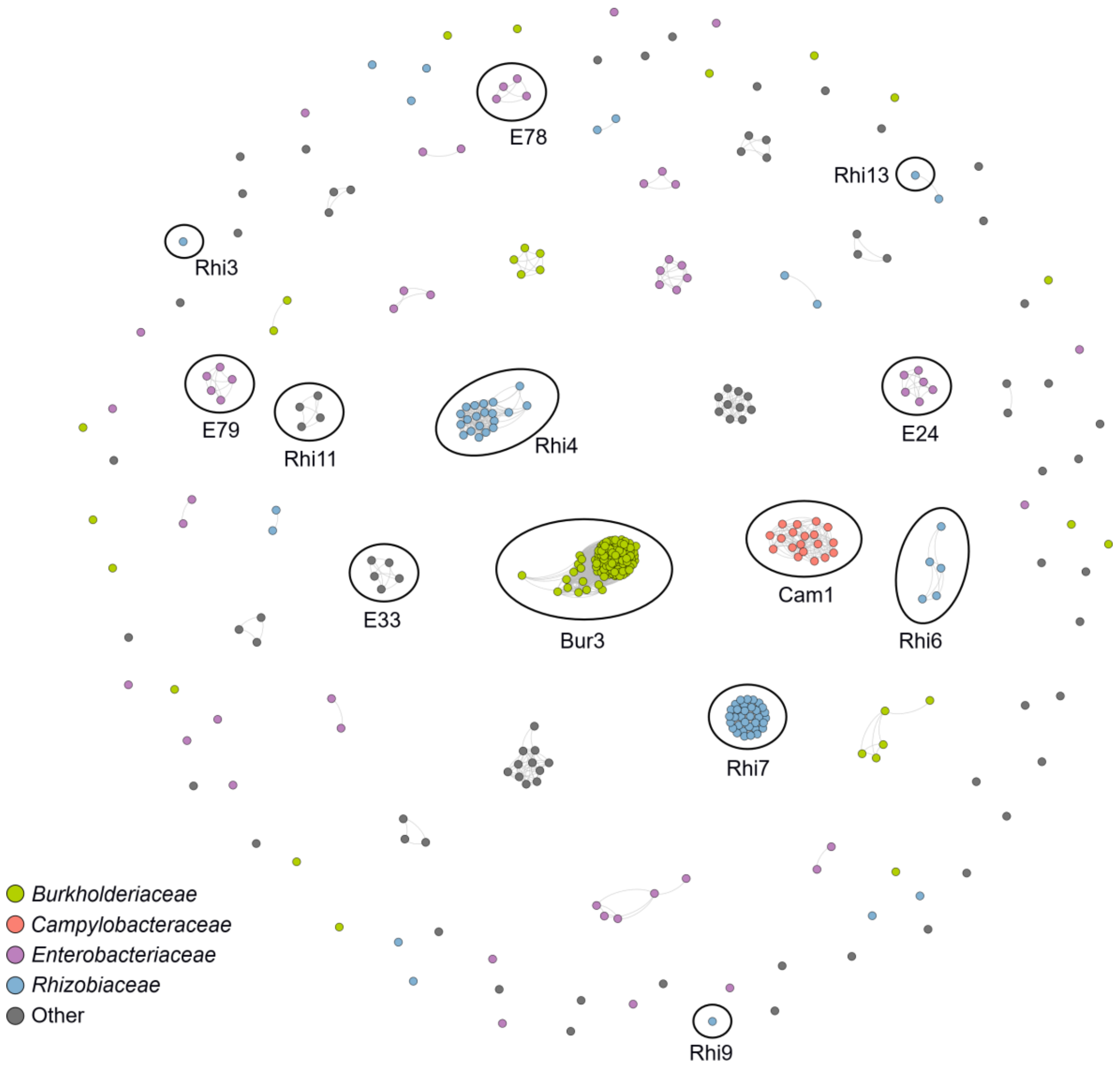

Supplementary Figure S2. ANI$_{L50}$ similarity network of plasmids encoding complete T6SSs. Nodes represent T6SS-encoding plasmids (n=375) and are colored according to the taxonomy of their hosts, as indicated in the legend. Plasmid Taxonomic Units (PTUs) are encircled when assigned.

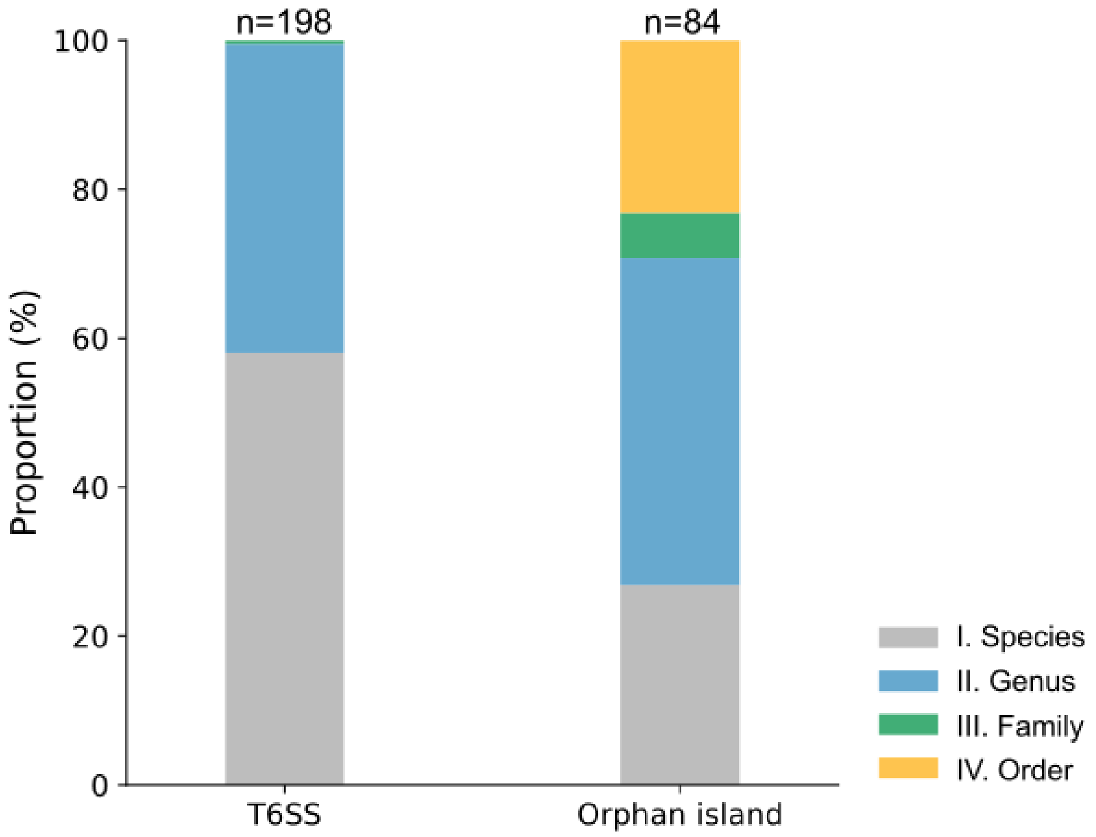

Supplementary Figure S3. Host range of PTUs encoding T6SS. Bar graph showing the taxonomic host distribution for PTUs encoding complete T6SSs (n=198) and orphan T6SS islands (n=84). Colors correspond to host taxa as indicated in the legend.

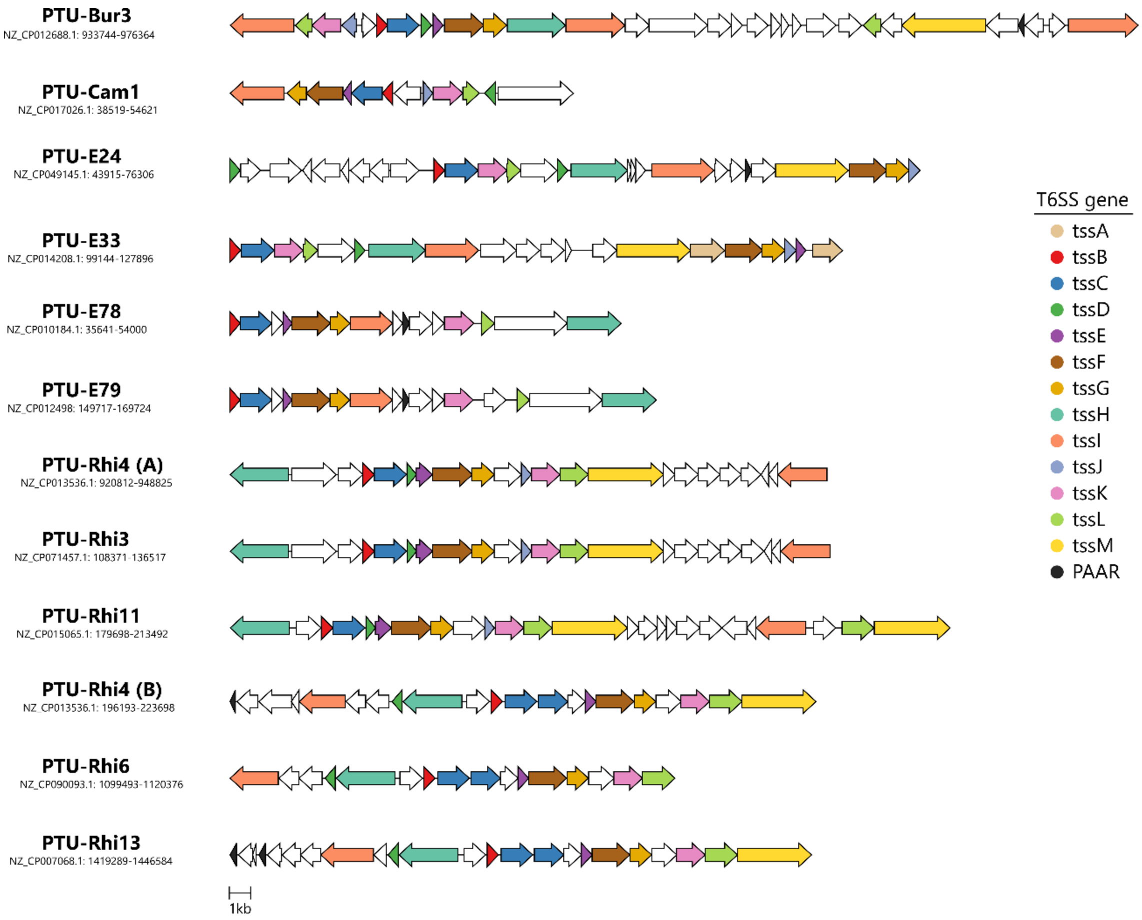

Supplementary Figure S4. T6SS genetic organization in representative plasmids from different PTUs. Synteny of complete T6SS loci is shown for each PTU using a representative plasmid genome (accession number and coordinates are indicated). T6SS genes identified in this study are colored according to the legend.

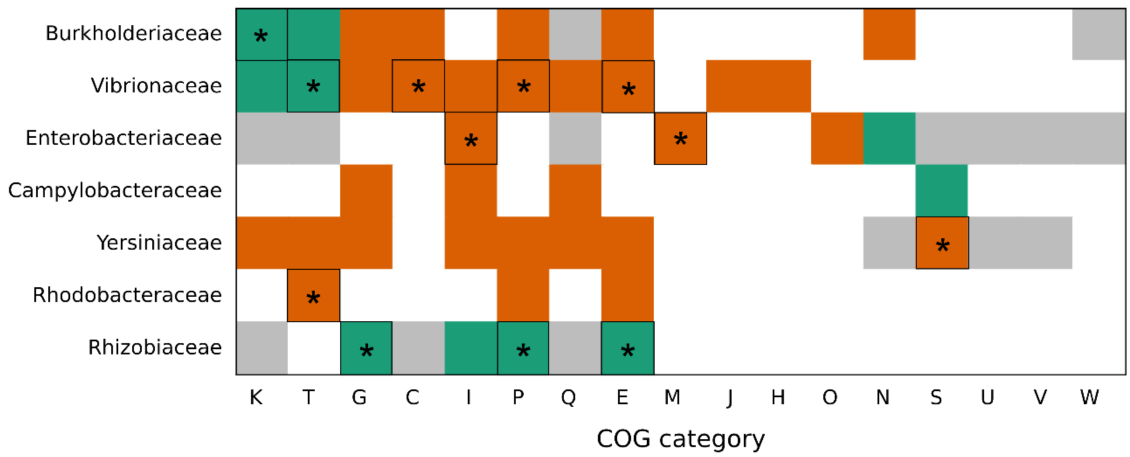

Supplementary Figure S5. Functional COG categories enriched in genomes encoding complete T6SS. For each host family, significantly enriched functional categories (Fisher's exact test with Benjamini-Hochberg correction, P_{adj} <0.05) are depicted as follows: orange if exclusive to plasmids, gray if exclusive to chromosomes, or green if present in both replicons. COG categories significantly enriched preferentially in T6SS^{+} megaplasmids relative to T6SS^{-} counterparts are marked with (*). List of COG categories: C: energy production and conversion; E: amino acid transport and metabolism; H: coenzyme transport and metabolism; I: lipid transport and metabolism; G: carbohydrate transport and metabolism; J: translation, ribosomal structure and biogenesis; K: transcription; M: cell wall/membrane/envelope biogenesis; N: cell motility; O: post-translational modification, protein turnover, chaperones; P: inorganic ion transport and metabolism; Q: secondary metabolites biosynthesis, transport and catabolism; S: function unknown; T: signal transduction mechanism; U: intracellular trafficking, secretion and vesicular transport; V: defense mechanisms; W: extracellular structures.

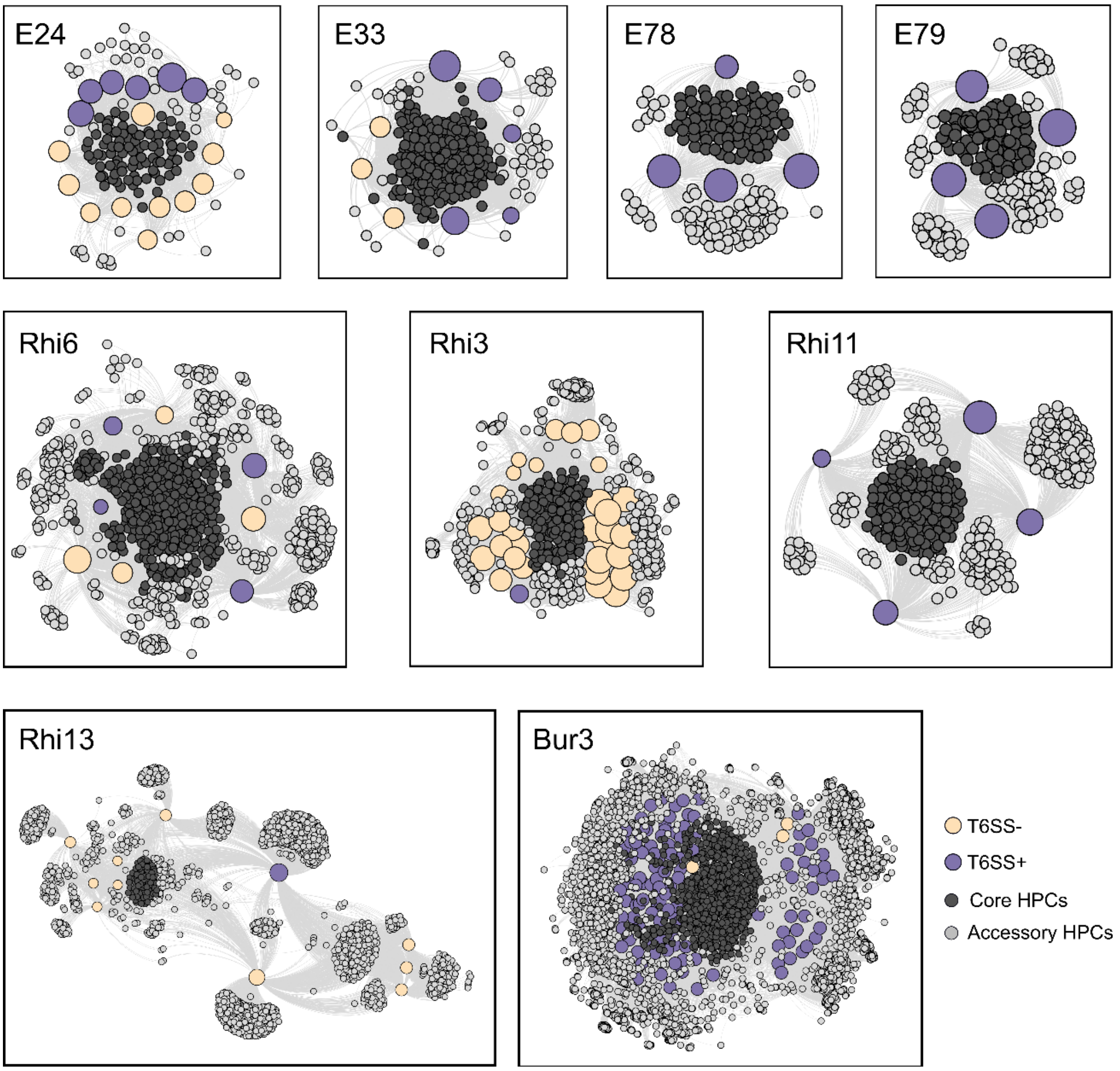

Supplementary Figure S6. Proteome networks of the T6SS-encoding PTUs. Bipartite proteome networks were generated with AcCNET at 80% identity and 80% coverage for each PTU containing plasmids encoding complete T6SS in this study. Large nodes represent plasmids colored according to the presence of T6SS[i]. Small nodes depict homologous protein clusters (HPC). HPCs corresponding to the core proteome (present in ≥ 80% of PTU members) are colored dark grey, while the remaining HPCs are colored light grey.

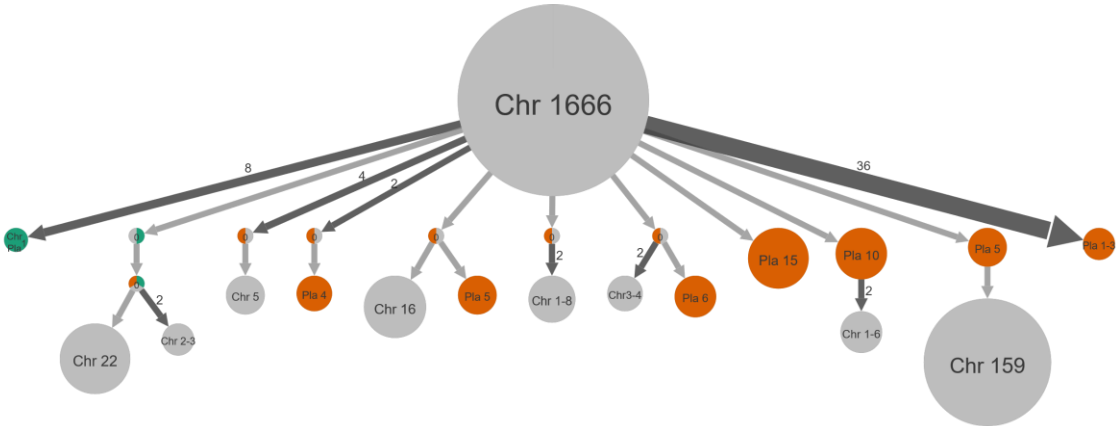

Supplementary Figure S7. Inference of ancestral states of TssC subunits. The ancestral states of TssC subunits from the phylogenetic tree (Figure 5) were inferred using PastML. Node colors indicate the TssC location: chromosomal T6SS in gray, plasmid-encoded TssC in orange and TssC found in both plasmid and chromosomal in green. Internal nodes shown as multi-colored circles containing “0” indicate ambiguous states for which no single ancestral state could be inferred. Numbers inside circles indicate the number of internal nodes or tree tips represented after vertical compression, whereas numbers on arrows indicate the number of identical subtrees merged horizontally. For a visual explanation of the interpretability of the ancestral compression, see the PastML help page (https://pastml.pasteur.fr/help).

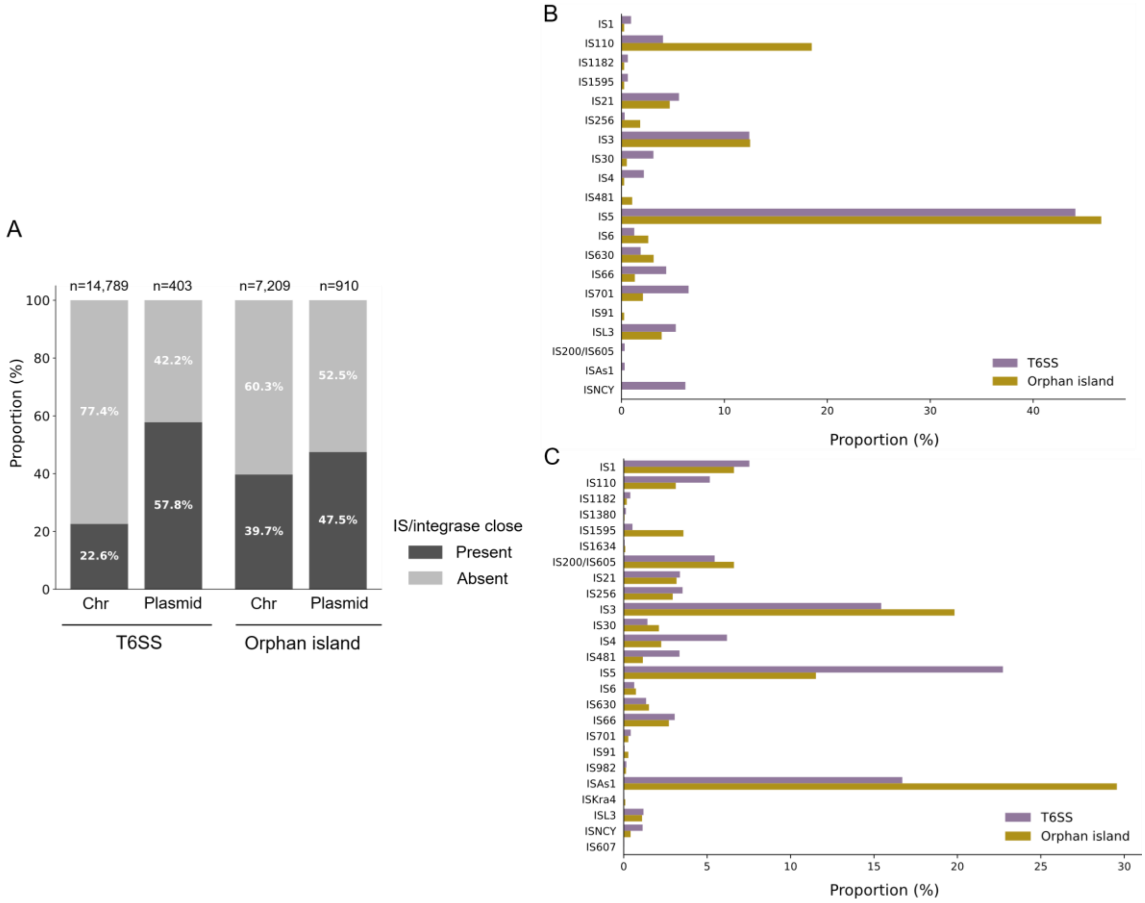

Supplementary Figure S8. Presence of insertion sequences (ISs) and integrases/recombinases in the proximity of T6SS^{i}. A) Proportion of complete T6SS^{i} clusters and orphan islands with an IS and/or integrase within 20 coding sequences from a T6SS component for chromosomes and plasmids. The number of T6SS^{i} clusters analyzed in each case is indicated. B) Abundance of each IS family near a T6SS^{i} or orphan island in plasmids. For each IS family, the proportion was calculated as the number of ISs of that family in close proximity relative to the total number of ISs detected near the corresponding cluster type (T6SS or orphan island). C) Abundance of each IS family near a T6SS^{i} or orphan island in chromosomes. The proportion was calculated as in B.

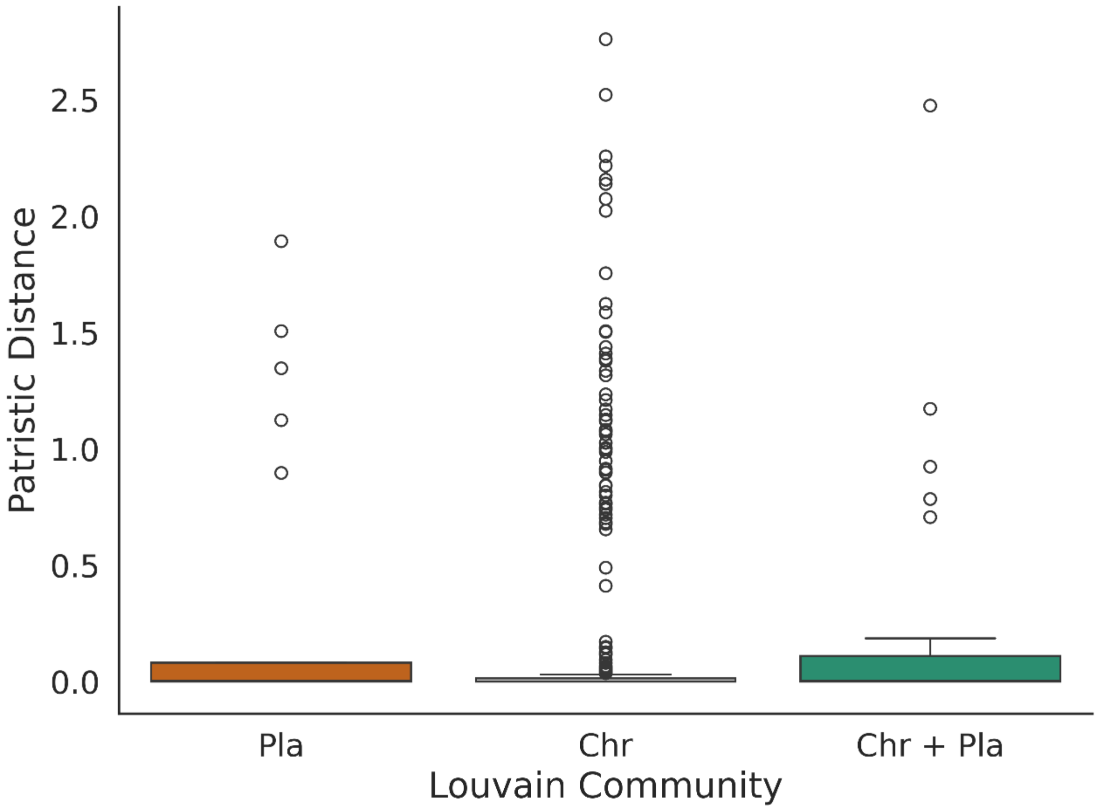

Supplementary Figure S9. Phylogenetic relationships among $T6SS^{i}$ in the horizontal gene transfer network. Distribution of patristic distances in the TssC tree (Figure 5A) for Louvain communities identified in the bipartite network (Figure 6A), considering T6SSs encoded exclusively on plasmids (Pla), exclusively on chromosomes (Chr), or on both plasmids and chromosomes (Chr + Pla).
